# DNA Backbone Sulfur-Modification Expands Microbial Growth Range under Multiple Stresses by its anti-oxidation function

**DOI:** 10.1038/s41598-017-02445-1

**Published:** 2017-06-14

**Authors:** Yan Yang, Guanpeng Xu, Jingdan Liang, Ying He, Lei Xiong, Hui Li, Douglas Bartlett, Zixin Deng, Zhijun Wang, Xiang Xiao

**Affiliations:** 1 State Key Laboratory of Microbial Metabolism, School of Life Science and Biotechnology, Shanghai Jiao Tong University, Shanghai, People’s Republic of China; 2 Central Analytical lab, School of Chemistry and Chemical Engineering, Shanghai Jiao Tong University, Shanghai, People’s Republic of China; 3 Center for Marine Biotechnology and Biomedicine, Scripps Institution of Oceanography, University of California, San Diego, La Jolla, CA USA

## Abstract

DNA phosphorothioate (PT) modification is a sulfur modification on the backbone of DNA introduced by the proteins DndA-E. It has been detected within many bacteria isolates and metagenomic datasets, including human pathogens, and is considered to be widely distributed in nature. However, little is known about the physiological function of this modification, and thus its evolutionary significance and application potential remains largely a mystery. In this study, we focused on the advantages of DNA PT modification to bacterial cells coping with environmental stresses. We show that the mesophile *Escherichia coli* and the extremophile *Shewanella piezotolerans* both expanded their growth ranges following exposure to extreme temperature, salinity, pH, pressure, UV, X-ray and heavy metals as a result of DNA phophorothioation. The phophorothioated DNA reacted to both H_2_O_2_ and hydroxyl radicals *in vivo*, and protected genomic DNA as well as sensitive enzymes from intracellular oxidative damage. We further demonstrate that this process has evolved separate from its associated role in DNA restriction and modification. These findings provide a physiological role for a covalent modification widespread in nature and suggest possible applications in biotechnology and biomedicine.

## Introduction

Modifications of DNA and RNA are involved in many physiological processes, including restriction-modification (R-M) systems and epigenetic control of DNA replication, transcription and translation^1^. DNA phosphorothioate (PT) modification is a novel modification on the DNA backbone, in which a non-bridging oxygen atom is swapped with a sulfur atom. It is a sequence-selective, stereospecific post-replicative modification governed by a family of proteins encoded by five genes, termed *dnd* (corresponding to the often-observed DNA degradation phenotype during electrophoresis)^2, 3^. DndA acts as a cysteine desulfurase and assembles DndC as an iron-sulfur cluster protein^4, 5^. In some cases, DndA can be functionally replaced by the cysteine desulfurase IscS^6^. DndB is a negative transcriptional regulator for the PT-modifying genes^7^, and is not essential for PT modification. DndC possesses ATP pyrophosphatase activity and is predicted to have PAPS reductase activity^5^. A DndD homolog known as SpfD in *Pseudomonas fluorescens* Pf0-1 has ATPase activity possibly related to DNA structure alteration or nicking during sulfur incorporation^8^. Structural analysis of DndE indicates that it is involved in binding nicked dsDNA^9^. However, the mechanisms of substrate recognition and the coordination of biochemical steps in PT synthesis are not known.

The presence of *dnd* genes and PT modifications has been established in over 200 phylogenetically diverse bacteria and archaea species, ranging from soil-inhabiting, antibiotic-producing *Streptomyces* species to human pathogens. Moreover, *dnd* gene homologues and PT modifications have also been detected in many metagenomes^10–15^. The widespread existence of PT modification implies a significant impact on bacteria, but the biological function of PT modification remains elusive. Recently, a 3-gene family was identified as a PT modification dependent restriction system^16^. About 86 PT modification strains possess these restriction gene homologues and about 125 strains lack the restriction system, indicating that *dnd*-encoded PT modifications provides functions other than R-M^17^. We propose that PT modification may play a role in microbial adaptation to many extreme conditions because many PT modification strains were isolated from environments with different stresses and our previous study shows that a PT modification strain exhibited advantageous antioxidation properties^18^. This hypothesis was first tested in an *E. coli* strain, and later extended to a deep-sea extremophilic bacterial strain.

## Results

### PT modification system endowed the *E. coli* strain Hpx^−^ growth advantages under multiple extreme conditions

**Figure 1:**
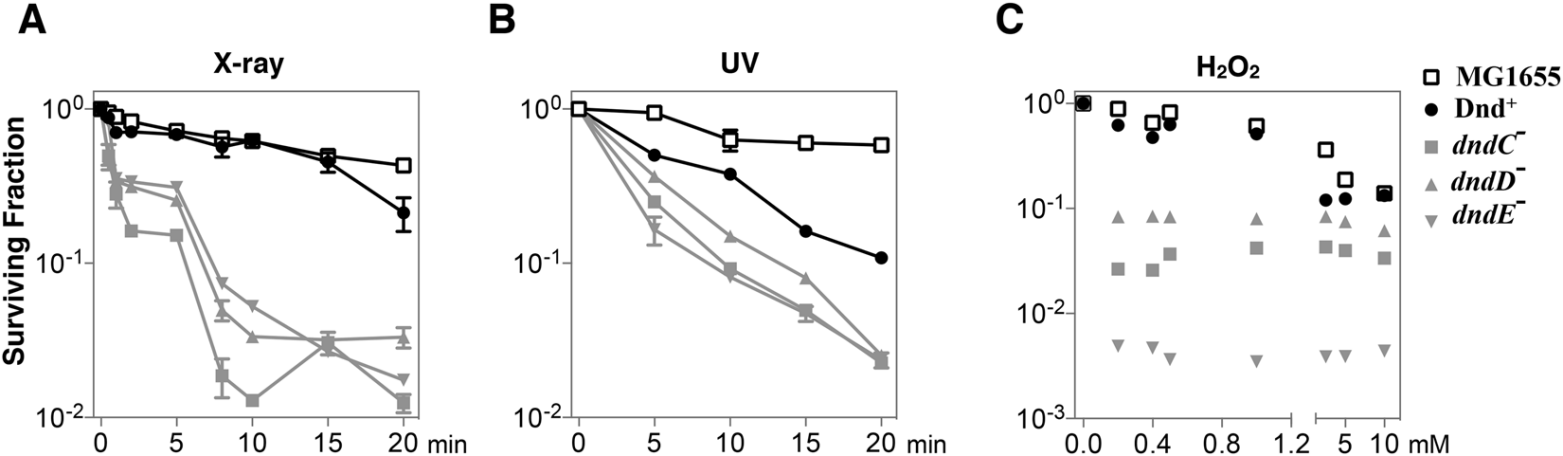
DNA PT modification enhances resistance to X-ray radiation, UV radiation and H_2_O_2_ exposure in an *E. coli* Hpx^−^ mutant. (**A**) The cells were treated with X-ray radiation for approximately 0 min, 2 min, 5 min, 8 min, 10 min, 15 min and 20 min and then plated to determine their viability. (**B**) The cells were treated with UV radiation for approximately 0 min, 5 min, 10 min, 15 min and 20 min and then plated to determine their viability. (**C**) The cells were treated with increasing concentrations of H_2_O_2_ for 15 min and then plated to determine their viability. The data shown represent the results of three independent experiments, and the error bars indicate the standard deviations.

**Figure 2:**
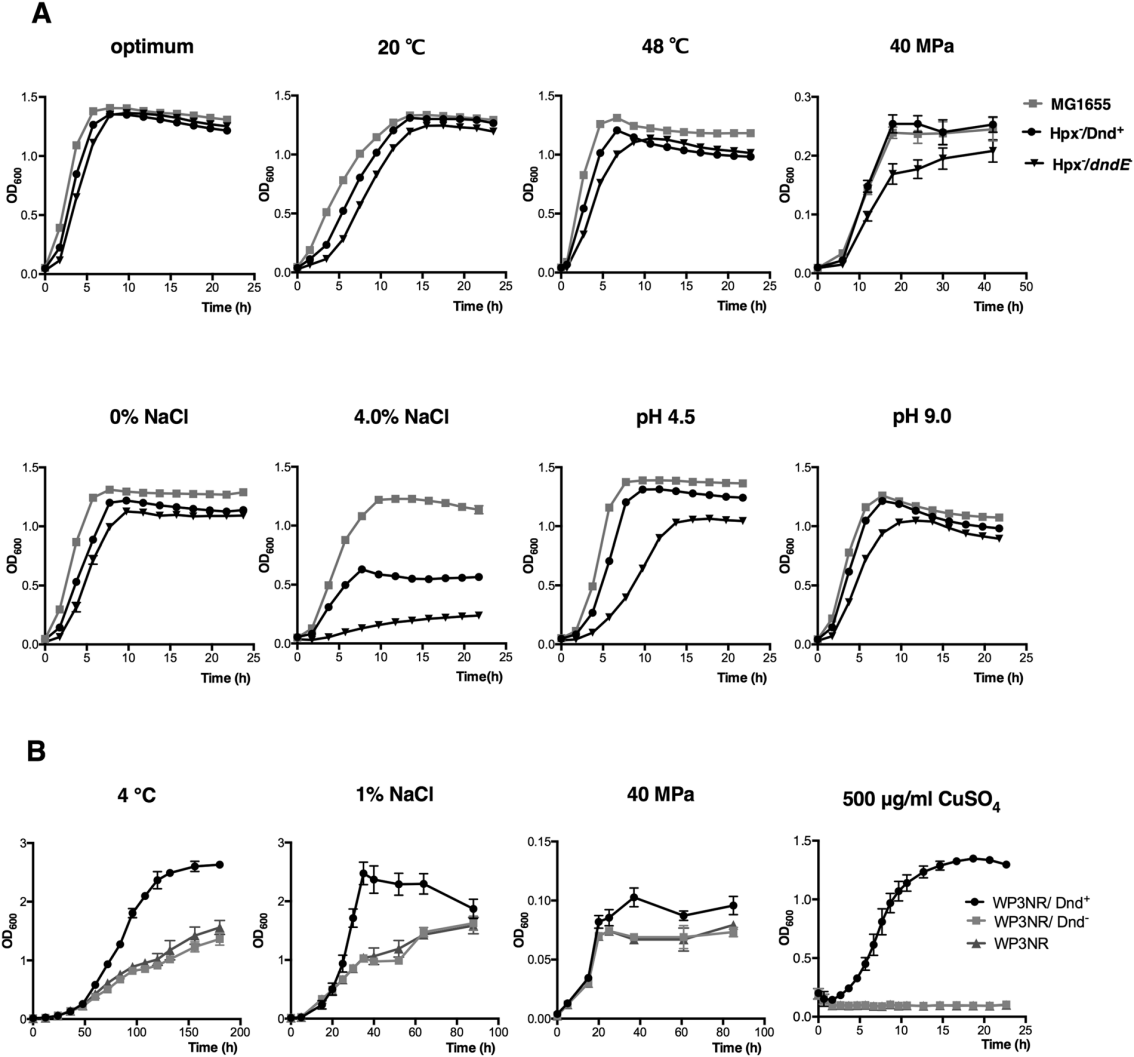
Growth of PT strains under different stress conditions. (**A**) Hpx^−^/Dnd^+^, wild-type *E. coli* strain MG1655, and Hpx^−^/*dndE* mutant cells were grown under optimal or temperature/pressure/salinity/pH/ CuSO_4_ stress conditions, and cell growth was monitored. (**B**) WP3NR/Dnd^+^, WP3NR/Dnd^−^ mutant and WP3NR cells were grown under low-temperature, low-salinity, high-pressure and CuSO_4_ stress conditions, and cell growth was monitored. The growth of the strains was detected at OD_600nm_. The data shown represent the results of three independent experiments, and the error bars indicate the standard deviations.

The plasmid containing the full-length *S. enterica dnd* gene cluster^3^ or the corresponding *dndC-E* in frame-deletion mutations^18^ were used to transform the *E. coli* catalase/peroxidase disruption strain Hpx^− ^^19^. Hpx^−^ mutants lack H_2_O_2_-scavenging capability and even anaerobic culturing generates sufficiently toxic levels of H_2_O_2_ to cause oxidative DNA damage^20^. DNA PT modification in the mutants was examined using the HPLC/MS method^14^. As expected, DNA PT modification was detected in Hpx^−^/Dnd^+^ but not in *dndC-E* deletion mutants (Supplementary Fig. 1). Hpx^−^/Dnd^+^ and its Dnd^−^ mutants (*dndC-E* mutants) and the *E. coli* wild-type strain MG1655 were then treated with X-ray radiation, UV radiation and H_2_O_2_. The survival rates markedly differed between the Dnd^+^ strain and the Dnd^−^ mutants in response to X-ray, UV and H_2_O_2_ treatments (Fig. 1), suggesting that DNA PT modification confers advantages under these stress conditions. The survival rates of Hpx^−^/Dnd^+^ were slightly lower comparing to those of MG1655 at these three conditions. The proliferation of Hpx^−^/Dnd^+^ cells exceeded that of the Dnd^−^ mutants in the presence of H_2_O_2_ (Supplementary Fig. 2A), and the growth rate of Hpx^−^/Dnd^+^ cells was comparable to that of MG1655 under H_2_O_2_ stress (Supplementary Fig. 2A and B). These observations show that the DNA PT modification system worked directly as an antioxidation system rather than by indirectly regulating the catalase/peroxidase genes, and the anti-oxidatant capablity of the PT modification strain Hpx^−^/Dnd^+^ is slightly weaker than that of the wild-type MG1655. The Hpx^−^/Dnd^+^, Hpx^−^/*dndE* deletion and MG1655 cells were further stressed with changes in pH, temperature, pressure, salinity and heavy-metal salts. Hpx^−^/Dnd^+^ strains exhibited superior proliferation under all tested extreme conditions except high temperature compared with Hpx^−^/*dndE* deletion strains (Fig. 2A; Supplementary Fig. 3; Supplementary Fig. 4A). Next, the Hpx^−^/Dnd^+^, Hpx^−^/*dndE* deletion and MG1655 cells were treated with three main classes of bactericidal antibiotics. Gentamicin was used to target translation, ampicillin to block cell-wall synthesis, and norfloxacin to disrupt DNA replication. To perform these tests, we cloned the *dnd* gene cluster and the corresponding *dndE* in frame-deletion mutation into a chloramphenicol-resistance plasmid and transferred them into Hpx^−^ cells. Hpx^−^/Dnd^+^ strains didn’t exhibit a survival advantage, suggesting that the PT modification system did not confer protection against antibiotic treatment (Supplementary Fig. 4B–D).

### PT modification system also endowed *Shewanella piezotolerans* WP3 growth advantages under multiple extreme conditions

To test the function of the PT system in an extremophile, a similar analysis was conducted in the deep-sea bacterium *Shewanella piezotolerans* WP3, a PT modification-free strain with an intact antioxidation system^21^ (Supplementary Table 1). *Shewanella piezotolerans* WP3 was isolated from sediment in the West Pacific at a depth of 1914 meters. It displays maximal growth at 20 °C, pH7, 20 MPa, and 3.4% NaCl^22^. WP3NR, a non-restricting strain derived from WP3, was constructed to facilitate *dnd* gene cluster transfer, and the identity of WP3NR/Dnd^+^ cells was verified using HPLC/MS (Supplementary Fig. 5). WP3NR/Dnd^−^, which is a WP3NR strain transformed with the empty vector, was used as a negative control. WP3NR/Dnd^+^ cells exhibited superior growth at low temperatures, high pressures, low salinity levels and heavy-metal stresses, but demonstrated a growth defect in stationary phase at high temperatures (Fig. 2B; Supplementary Figs 6, 7). This result demonstrated that the lateral transfer of a PT modification system remained effective when overlapped with the existing antioxidation system, especially under specific conditions.

### PT modification system protected both DNA and protein *in vivo*

**Figure 3:**
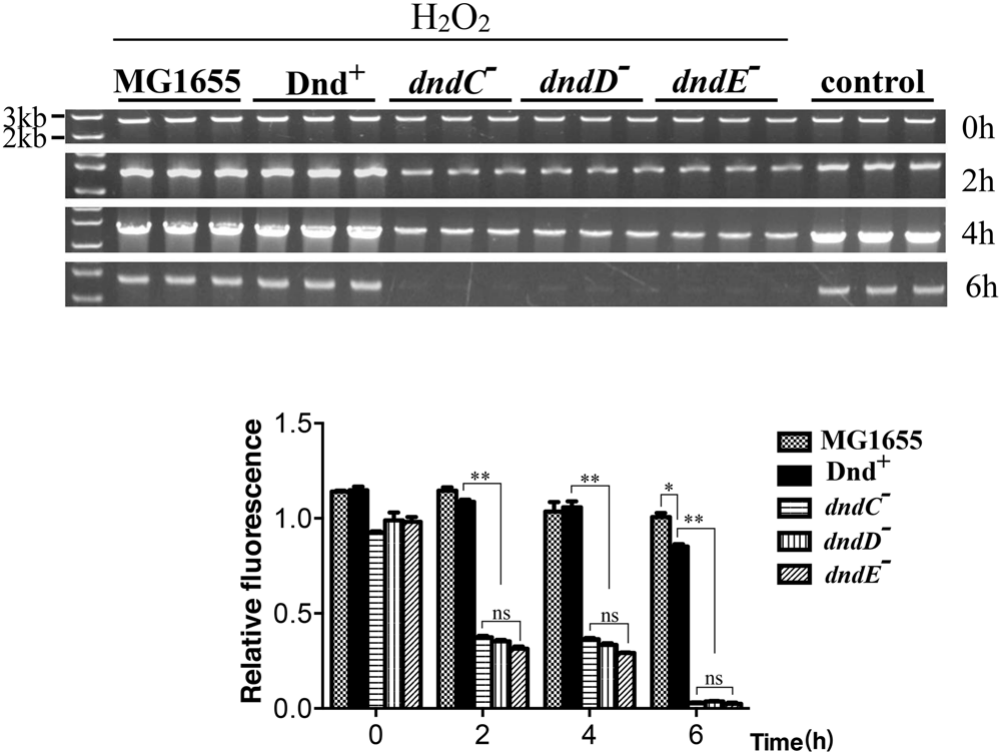
DNA phosphorothioation protects Hpx- cells from oxidative damage to genomic DNA. MG1655 and Hpx^−^/Dnd cells were treated with 1 mM H2O2. The reaction was stopped at 2-hour intervals by adding catalase, and total genomic DNA was isolated and subjected to polymerase chain reaction (PCR) amplification to obtain a 2.7 kb DNA fragment. PCR was performed using equivalent amounts of template DNA for each reaction mixture. The PCR products were fractionated and scanned on an agarose gel to assess DNA oxidative damage. The loading quantities of PCR products were adjusted properly in order to observe the bands of Hpx^−^/*dnd* mutants, and the amounts of DNA were normalized to that of the untreated control (Hpx^−^). Values are the means and standard deviations from three experiments. The data were analyzed by Student’s t test. **P < 0.01; ns, not significantly different. Full-length gels are included in Supplementary Figure 9.

In many extreme conditions, excessive reactive oxygen species (ROS) are generated^23–25^. To determine whether PT modification can protect genomic DNA from oxidative damage *in vivo*, *E. coli* Hpx^−^/Dnd^+^ and Hpx^−^/*dnd* mutant and MG1655 cells were challenged with 1 mM H_2_O_2_. Next, genomic DNA was isolated and oxidative damage was monitored using PCR analysis. The DNA samples isolated from Hpx^−^/Dnd^−^ mutants (*dndC-E* mutants) were all damaged by H_2_O_2_ obviously, but DNA containing the PT modification was less damaged than the modification-negative counterpart (Fig. 3). Moreover, the DNA damage level only markedly differed at the 6-hour sampling time between Hpx^−^/Dnd^+^ and MG1655. This result shows that the DNA PT modification can function as a self-defense system and protect DNA from oxidative damage.

**Figure 4:**
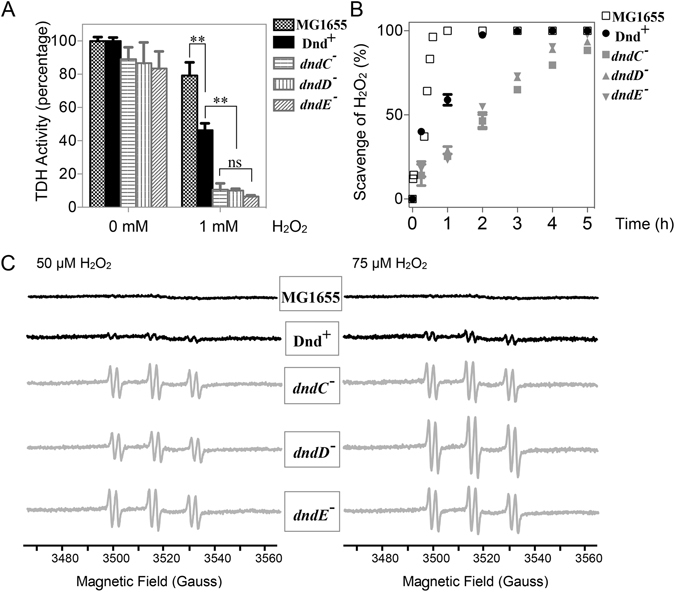
DNA phosphorothioation protects TDH enzyme and scavenge H_2_O_2_, hydroxyl radicals *in vivo*. (**A**) To assess the damage to TDH, the cells were treated for 20 minutes and total protein was extracted; TDH activity was then anaerobically assayed. The data shown represent the results of three independent experiments, and the error bars indicate the standard deviations. The data were analyzed by Student’s t test. **P < 0.01; ns, not significantly different. (**B**) MG1655 and Hpx^−^/Dnd whole cells were incubated with 1 mM H_2_O_2_, and the disappearance of H_2_O_2_ was monitored. The data shown represent the results of three independent experiments, and the error bars indicate the standard deviations. (**C**) Hydroxyl radicals in MG1655 and Hpx^−^/Dnd cells were induced by adding 50 or 75 μM exogenous H_2_O_2_. The signals were detected using POBN-ethanol spin trapping. The cell densities were equivalent in all samples. The data are representative of three independent experiments. The six peaks represent the hydroxyl radical signals.

To test whether PT modification can also protect other biomolecules, threonine dehydrogenase (TDH) was selected as a reporter biomolecule. TDH is a representative of a family of mononuclear enzymes that includes epimerases, dehydrogenases, deformylases and deaminases, which are highly sensitive to H_2_O_2_. As little as 0.5 μM H_2_O_2_ can inactivate these enzymes, causing the release of Fe^3+^ via the Fenton reaction between H_2_O_2_ and iron^26^. After treating Hpx^−^/Dnd^+^ and Hpx^−^/*dnd* mutant and MG1655 cells with 1 mM H_2_O_2_ for 15 minutes, the total proteins were extracted, and TDH activities were assayed anaerobically. TDH from MG1655 strain lost about 20% of its total activity, as shown in Fig. 4A. TDH from the Hpx^−^/ Dnd^+^ strain lost about 50% of its total activity, whereas the enzyme from the Hpx^−^/*dnd* mutant strains lost approximately 90% of its activity. This demonstrates that the modification not only protects genomic DNA but also protects enzymes from oxidative damage, with a potential function as a comprehensive antioxidation system.

### PT modification system scavenged H_2_O_2_ and hydroxyl radicals ***in vivo***

This idea was further tested *in vivo* using the *E. coli* Hpx^−^/Dnd^+^ and Hpx^−^/*dnd* mutant and MG1655 cells. Bacterial cells that had reached an OD_600_ of 1.2 were challenged with 1 mM H_2_O_2_, and the disappearance of H_2_O_2_ in the LB medium was then monitored. H_2_O_2_ disappeared in all strains because of the scavenging activity of the rich LB medium and antioxidants inside the cells, but H_2_O_2_ was scavenged more rapidly in the Dnd^+^ strain (Fig. 4B). Therefore, the PT modification system can scavenge hydrogen peroxide *in vivo*. Hydroxyl radicals in bacterial cells can be trapped as hydroxyethyl radical spin adducts and analyzed using spin trapping electron paramagnetic resonance (EPR)^27^. Treatment with 50 μM H_2_O_2_ markedly increased adduct formation in Hpx^−^/*dnd* mutant cells compared with Dnd^+^ cells and MG1655 cells (Fig. 4C). The magnitude of the signals increased when 75 μM exogenous H_2_O_2_ was added. However, only very weak signals were detected for the Dnd^+^ strain when compared with the *dnd* mutants (Fig. 4C). This result shows that DNA PT modification also scavenges hydroxyl radicals *in vivo*.

### Evolutionary implications of bacterial strains with PT modification system

After searching all 47,246 publicly available bacterial genomes in NCBI, a total of 836 genomes containing *dnd* gene clusters were identified, 390 of which were also found to contain a restriction system (*dptFGH*). The remaining 446 genomes only contained *dnd* gene clusters (Supplementary Table 2). Meanwhile, a total of 50 genomes were found to only contain *dptFGH* gene clusters without *dnd* gene clusters, and this number may be underestimated due to the missing prediction and/or annotation of *dnd* gene clusters. Based on the distribution patterns of *dnd* and *dpt* gene clusters among bacterial phylogenic groups (Supplementary Fig. 8) it is evident that the *dnd* gene clusters are more widespread and located in deeper branches of the Bacteria domain than is the case for genomes harboring both the *dnd* and *dpt* gene clusters. This observation suggests that the PT modification system originated as an antioxidation system and later developed into an atypical restriction-modification system.

## Discussion

A variety of experimental approaches have demonstrated that various kinds of stresses exert some of their deleterious effects by amplifying the natural rate of ROS production. Heat, desiccation and ionizing radiation all result in increased ROS levels^28–30^. In this study, we have discovered that the PT modification system can scavenge hydrogen peroxide and hydroxyl radicals *in vivo* and protect both genomic DNA and proteins from oxidative damage. PT modified strains of *E. coli* Hpx^−^ and *S. piezotolerans* WP3 didn’t exhibit growth/survival advantages under some of the environmental stresses tested, like high temperature and antibiotic treatment. The effect of PT modification on the responses to these factors might vary among strains and species. It has been reported that high temperature can induce oxidative stress^31, 32^ and reactive species including ROS generated by antibiotic treatment contribute to antibiotic lethality^33–35^. In the H_2_O_2_ scavenging assay, considering there are about 5 × 10^3^ copies of PT modifications in one *E. coli* cell^14^, the number of H_2_O_2_ molecules consumed in an hour was ~3–4 orders higher than the total thioate content (about 10 μM/per cell). This indicates that an unknown antioxidation mechanism exists which functions in a non-stochiometric fashion. This process awaits future elucidation. Based on our combined experimental results, PT modification system could not restore the entire antioxidant capability of the wild-type MG1655, but it could significantly improve (>50%) the antioxidant capablility and the viability of Hpx^−^ cells.

In this work, we show that DNA PT modification functions as a common strategy used by bacterial cells to cope with different kinds of environment stresses. Exposure to ROS present in nature, such as in macrophages, biofilms, chlorine-treated wastewater and extreme environments, would enhance the spread of this novel antioxidation system. Our findings might help to understand the reasons underlying the widespread existence of this system in pathogens such as *Clostridium difficile* ^36^, which is catalase-deficient. Moreover, nearly half of US clinical isolates of the emerging pathogen *Mycobacterium abscessus* were reported to exhibit smeared DNA during pulsed field gel electrophoresis, also suggesting that the Dnd phenotype is common in this pathogen^11^. The finding that PT modified DNA is highly efficient at removing ROS warrants further examination in the context of its application in biological engineering and therapeutic applications: Redox reactions have significant commercial utility, as they play a key role in the synthesis of industrially relevant compounds. Nucleoside PT derivatives have already been shown to be highly effective neuroprotectants in the treatment of Alzheimer’s disease^37, 38^. Further understanding the mechanism underlying PT modification could help to cure diseases related to ROS accumulation, such as cancer and neurodegenerative diseases. However, it should be carefully evaluated because a recent report described that PT oligonucleotides caused specific platelet activation^39^, which may limit its therapeutic applicability.

## Materials and Methods

### Chemicals, enzymes, and media

Hydrogen peroxide, ferric sulfate, ferric chloride, catalase, the hydrogen peroxide assay kit, and the phosphorothioate-modified dinucleotides dGsA and dGA were purchased from Sangon Biotech (Shanghai) Co., Ltd. Trimethoprim, thymine, α-(4-pyridyl-N-oxide)-N-tert-butylnitrone (POBN), diethylenetriaminepentaacetic acid (DTPA), Hanks’ Balanced Salt Solution (HBSS, without phenol red, calcium chloride and magnesium sulfate), threonine, NAD^+^ and Chelex-100 were purchased from Sigma. The DNeasy Tissue kit was purchased from QIAGEN. The Cycle-Pure Kit, Bacterial DNA Kit, and Gel Extraction Kit were purchased from OMEGA Bio-Tech. Luria-Bertani (LB) broth contained 10 g/L of BactoTryptone, 5 g/L yeast extract, and 10 g/L sodium chloride unless otherwise noted. The modified marine broth medium 2216E contained 5 g/L tryptone, 1 g/L yeast extract, 0.1 g/L FePO_4_, and 34 g/L sodium chloride. K medium contained A salts, 0.2% glucose, 1 mM MgCl_2_, 0.5 mM amino acids and 5 mg/L thiamine. The media were supplemented with the following concentrations of antibiotics (unless otherwise noted): ampicillin (Amp) 100 μg/ml and chloramphenicol (Cml) 12.5 μg/ml. Solid medium was supplemented with 1.5% (w/v) agar-A.

### Strains, plasmids and growth conditions

The strains and plasmids used in this study are listed in Supplementary Table 3. The *E. coli* strains were grown at 37 °C, and the *Shewanella* strains were grown at 20 °C. Aerobic cultures were grown in tubes or flasks shaking at a speed of 220 rpm; anaerobic cultures of *E. coli* strains were grown in test tubes in an anaerobic box containing 90% N_2_ –10% H_2_ gas at room temperature. *E. coli* strain WM3064 was incubated at 37 °C in LB broth containing 50 μg/ml DL-α, ε-diaminopimelic acid (DAP). High-pressure cultivation was performed using a hand-operated pump and a quick-fit connector attached to the high-pressure vessels. Overnight log phase cultures were diluted 100-fold with the same medium, transferred to polyethylene bulbs, sealed without an air space and placed inside pressure vessels. Pressure was applied using a hand-operated pump and a quick fit connector attached to the pressure vessel^40^.

### Construction of plasmids

DNA fragments coding for the *dndA* and *dndBCDE* genes were amplified by PCR using plasmids pJTU3619 and pJTU1238 as templates, respectively. The *dndA* products were cloned between *Xho*I and *Kpn*I into the corresponding sites of pSW2. The *dndBCDE* products were then cloned between *Pst*I and *Kpn*I into the corresponding sites, yielding pSW2Dnd^+^. The same strategy was used to construct other plasmids. To construct a gene in-frame deletion plasmid, fusion PCR was performed as described previously^41^. All plasmids were confirmed by sequence analysis.

### Construction of DNA PT modification strains

For *Shewanella piezotolerans* strains, the plasmid pSW2Dnd^+^ was transformed into *E. coli* WM3064 and then into WP3NR by two-parent conjugation. For *E. coli* strains, plasmids were introduced by calcium chloride transformation. DNA PT modification in the mutants was detected using HPLC/MS methods described below.

### Bacterial growth curves under extreme conditions

Temperature, hydrostatic pressure, salinity and pH ranges for growth were tested before determining the growth curve for both *Shewanella piezotolerans* and the *E. coli* strains. The following conditions were selected as extreme conditions for *Shewanella piezotolerans* strains: temperature (4 °C, 28 °C), salinity (1% NaCl, 5.1% NaCl), hydrostatic pressure (40 MPa), pH (6.0, 10.0) and heavy-metal salts (500 μg/ml CuSO_4_; 500 μg/ml ZnSO_4_; 500 μg/ml MnCl_2_). The following conditions were selected as extreme conditions for *E. coli* strains: temperature (20 °C, 48 °C), salinity (0% NaCl, 4.0% NaCl), hydrostatic pressure (40 MPa), pH (4.5, 9.0) and heavy-metal salts (500 μg/ml CuSO_4_; 200 μg/ml ZnSO_4_; 5.0 mg/ml MnCl_2_). Overnight log-phase cultures of *Shewanella* cells were diluted approximately 100-fold with the same medium to the starting OD_600_ value, and OD_600_ was measured over time. And WP3 cells were diluted approximately 20-fold with the same medium to the starting OD_600_ value in heavy-metal salt medium, and the growth curves were monitored using Bioscreen C from Bioscreen. The *E. coli* Hpx^−^/Dnd strains were cultured in LB medium supplemented with catalase (100 U/ml) at 37 °C to mid-log phase. The cultures were centrifuged at 4,000 × g for 2 min, and cell pellets were washed three times with fresh LB. Washed cells were resuspended in the corresponding medium, diluted approximately 100-fold to the starting OD_600_, and the OD_600_ was measured over time. And cells were diluted approximately 20-fold with the same medium to the starting OD_600_ value in heavy-metal salt medium. The growth curves of *E. coli* strains were monitored using Bioscreen C from Bioscreen, and 20 °C was selected as the low-temperature stress due to instrument limitations.

### Bacterial growth curves with H_2_O_2_ treatment

The *E. coli* Hpx^−^/Dnd strains and the wild type MG1655 were aerobically cultured in LB medium at 37 °C overnight. The cultures were then diluted to an OD_600_ of 0.1 in LB. H_2_O_2_ was added to final concentrations of 0 mM, 0.2 mM, 0.4 mM, and 0.8 mM. The growth curves were monitored using BioTek^®^SynergyII from Gene Co.Ltd. The OD_600_ was monitored for 8 hours at 30 minute intervals. *E. coli* MG1655 and Hpx^−^/Dnd^+^ were aerobically cultured in LB at 37 °C in the same manner. The cultures were then diluted to an OD_600_ of 0.1 in LB at 37 °C. H_2_O_2_ was added to final concentrations of 0 mM, 1 mM, 2.5 mM, and 5 mM. The growth curves were monitored at OD_600_ for 8 hours at 30 minute intervals.

### UV killing assay

Bacterial cells were grown aerobically at 37 °C overnight. The cultures were then diluted to an OD_600_ of 0.1, and the cells were then plated on plates and placed under UV light for 0 min, 5 min, 10 min, 15 min, and 20 min on clean benches. The cultures were then diluted with LB medium and plated on Luria Broth agarose plates. The plates were incubated overnight at 37 °C. The colonies were counted to determine the viable cells.

### X-ray killing assay

Bacterial cells were aerobically grown over night at 37 °C. The cultures were diluted to an OD_600_ of 0.1 and then exposed to X-ray irradiation from an X-ray detector (COMET, MXR-160HP/11, 160 kV, 6 mA) for 0 min, 30 s, 1 min, 2 min, 5 min, 8 min, 10 min, 15 min, and 20 min. The cultures were diluted with LB medium and plated on Luria Broth agarose plates. The plates were incubated overnight at 37 °C, and the colonies were counted to determine the viable cells.

### H_2_O_2_ killing assay

Bacterial cells were grown aerobically at 37 °C overnight. The cultures were diluted to an OD_600_ of 0.1 in K medium and grown to an OD_600_ of 0.8 at 37 °C. H_2_O_2_ was added to a final concentration of 0 mM, 0.2 mM, 0.4 mM, 0.5 mM, 1 mM, 2.5 mM, 5 mM and 10 mM, then cells were incubated for 15 min at 37 °C. Catalase (2,500 U/ml) was added to terminate the reaction. The cultures were diluted with LB medium and plated on Luria Broth agarose plates. The plates were incubated overnight at 37 °C, and the colonies were counted to determine the viable cells.

### Antibiotic killing assay

The antibiotic killing assay was performed as described by Kim Lewis *et al*. without modification^42^. Overnight cultures were diluted 1:500 into 4.5 ml of LB broth/2216E medium in 30 ml in bottles and grown for 2.5 hrs at 37 °C in a shaking incubator (300 rpm). After addition of antibiotic, the cultures were returned to the shaker for 3 hrs. The cultures were diluted with LB broth/2216E medium and plated on LB broth/2216E medium agarose plates. The plates were incubated at 37 °C/20 °C, and cell survival was determined by colony count.

### DNA damage quantification using PCR

*In vivo* DNA damage was characterized according to methods described by Sunny Park *et al*.^27^. Cells were aerobically grown at 37 °C, and the cultures were diluted to an OD_600_ of 0.1 in LB before the addition of 1 mM H_2_O_2_. The cultures were aerobically grown at 37 °C, and 5 ml samples were withdrawn at two-hour intervals to extract genomic DNA using the DNeasy Tissue kit (Qiagen). The extracted DNA was quantified using a NANODROP 2000 spectrophotometer (Thermo). The following primer sequences were used to amplify a 2.7-kb DNA fragment: 5′-TGCTGGAGCA ACTGAAGCGT (forward primer) and 5′-TGGCACCGAGACAACCAGCT (reverse primer). PCR was performed using the rTaq PCR system (Takara). The 25 μl PCR mixture contained 0.01 ng to 0.08 ng of genomic DNA as the template, 2 μM of the two primers, 200 μM dNTP mixture (Takara), 10 × PCR buffer, and 0.5 μl of the polymerase. The PCR conditions were set to the following: denaturation for 5 min at 94 °C, followed by 22 cycles of amplification, which consisted of denaturation at 94 °C for 30 seconds, annealing at 60 °C for 30 seconds and extension at 72 °C for 3 minutes. A final extension step at 72 °C was performed for 10 minutes. The products were separated on a 1% agarose gel by electrophoresis, stained using ethidium bromide, scanned on a BIO-RAD Gel DocTMX^®^, and quantified with ImageLab^TM^ Software.

### H_2_O_2_ scavenging by whole cells

Cultures were grown aerobically overnight at 37 °C in LB that contained 100 unit/ml catalase. The cells were washed twice with 5 ml of LB medium to remove catalase and finally diluted to an OD_600_ of 0.4 in LB and 1 mM H_2_O_2_ was then added. The cells were incubated at 37 °C. At one-hour intervals, 1 ml samples were withdrawn and centrifuged at 12,000 rpm for 1 min to remove the bacterial cells. The resultant supernatants were then diluted using water to determine the concentration of H_2_O_2_ using the Ferric–Xylenol orange peroxide assay without modification^43^.

### Threoninedehydrogenase (TDH) activity assays

Total TDH activity was assayed as described by Anjem *et al*. without modification^44^. Cultures were aerobically grown overnight at 37 °C in LB containing 100 U/ml catalase. The cells were then washed three times with 5 ml of pre-cold HBSS without catalase. The cells were then treated with 0 μΜ or 1 mΜ H_2_O_2_ for 15 min, followed by three washes with HBSS. The cells were then transferred to an anaerobic chamber, resuspended in Tris-HCl buffer (50 mM Tris-HCl, pH 8.4) and quickly sonicated. TDH activities were then immediately anaerobically assayed in a 500 μl reaction mixture containing 50 mM Tris-HCl buffer at pH 8.4, 1 mM NAD^+^, 30 mM threonine, 100 μΜ ferrous iron, and the extracted total protein. The reactions were stopped by adding 25 μΜ nitrobenzoic acid, and TDH activities were measured at 340 nm. The total protein content was determined using the Coomassie Blue dye binding assay (TIANGEN BIOTECH CO. LTD.).

### Spin trapping of hydroxyl radicals *in vivo*

The hydroxyl radical signal was trapped *in vivo* according to protocols described by Lee Macomber *et al*.^45^. The cultures were aerobically grown overnight at 37 °C in LB containing 100 U/ml catalase. The cultures were then diluted to an OD_600_ of 0.2 and grown aerobically at 37 °C until reaching an OD_600_ of 0.4. The medium was removed by centrifugation. The bacterial cell pellets were washed three times using ice-cold Chelex-treated HBSS. Approximately 100 μl of cell suspension was added to a 100 μl mixture of 100 μΜ DTPA, 10 mM 4-POBN, 170 mM ethanol, 50 μΜ or 75 μΜ H_2_O_2_ and treated HBSS at room temperature. The final strain densities (OD_600_ = 20) were equivalent in all samples. The spectra were measured using a Bruker EMX-8 after 15 min at room temperature with the following settings: field center, 3515 G; field sweep, 100 G; modulation frequency, 100 kHz; modulation amplitude, 1 G; time constant, 0.051; receiver gain, 50200; and power, 20 mW.

### Liquid chromatography mass spectrometry (HPLC/MS) analysis

DNA PT modifications were analyzed using HPLC/Mass Spectrometry (Agilent 1100 series LC/MSD Trap system or Agilent HPLC 1290-MS 6230 system) *(8)*. Briefly, modified DNA was digested using nuclease P1 and alkaline phosphatase. Twenty microliters of reaction products were loaded on a YMCC18 reverse-phase column (250 × 4.6 mm, 5 μm) and detected at 254 nm. HPLC was carried out at a flow rate of 0.3 ml/min at room temperature. Buffer A consisted of 0.1% acetic acid in water, and buffer B consisted of 0.1% acetic acid in acetonitrile. The following HPLC gradient was employed: 1–13% buffer B for 30 min, 13–30% buffer B for another 15 min, followed by 30–1% buffer B for 5 min, and finally 1% buffer B for 15 min. The drying gas flow was 10 L/min, the nebulizer pressure was 30 psi, the drying gas temperature was 325 °C, and the capillary voltage was 3,200 V (using the positive detection mode and scanning between 50 and 1000 m/z for mass spectrometer analysis).

### Identification and phylogenetic analysis of the *dnd* and *dpt* clusters

The identification of Dnd protein sequences was based on the TIGR database (http://www.tigr.org) annotation, i.e., TIGR03235, TIGR 03233, TIGR03183, TIGR03185 and TIGR03184 for DndA, B, C, D, and E, respectively. All publicly available bacterial genomes from the NCBI database (http://www.ncbi.nlm.nih.gov/genome) were scanned for the presence of the *dnd* cluster. The sequences of selected bacterial 16 S rRNA genes were aligned using ClustalW^46^ and then subjected to a maximum likelihood analysis in FastTree^47^. To identify the presence of the *dnd* cluster, the genomic coordinates and gene order of all annotated *dnd* genes were manually checked. Genomes with successive *dndCDE* were considered to be positively detected for the *dnd* gene cluster. A similar approach was applied to identify the *dpt* cluster, which was characterized by the presence of *dptF*, *dptG*, and *dptH*.

## Electronic supplementary material

supplementary information
