# Supplementary material for: DNA Backbone Sulfur-Modification Expands Microbial Growth Range under Multiple Stresses by its anti-oxidation function
Source: Sci Rep. 2017 Jun 14;7:3516. doi: 10.1038/s41598-017-02445-1 (PMC5471199; doi:10.1038/s41598-017-02445-1)
Supplement: Supplementary file 1 — supplementary information [file 41598_2017_2445_MOESM1_ESM.pdf]

DNA Backbone Sulfur-Modification Expands Microbial Growth Range under Multiple Stresses by its anti-oxidation function

Authors: Yan Yang[1†], Guanpeng Xu[1†], Jingdan Liang[1†], Ying He[1], Lei Xiong[1], Hui Li[2], Douglas Bartlett[3], Zixin Deng[1], Zhijun Wang[1*], and Xiang Xiao[1*]

Affiliations:

[1]State Key Laboratory of Microbial Metabolism, School of Life Science and Biotechnology, Shanghai Jiao Tong University, Shanghai, People's Republic of China.

[2]Central Analytical lab, School of Chemistry and Chemical Engineering, Shanghai Jiao Tong University, Shanghai, People's Republic of China.

[3]Center for Marine Biotechnology and Biomedicine, Scripps Institution of Oceanography, University of California, San Diego, La Jolla, CA, USA.

*Corresponding author. E-mail: wangzhijun@sjtu.edu.cn; xoxiang@sjtu.edu.cn.

†These authors contributed equally to this work.

Supplementary Materials

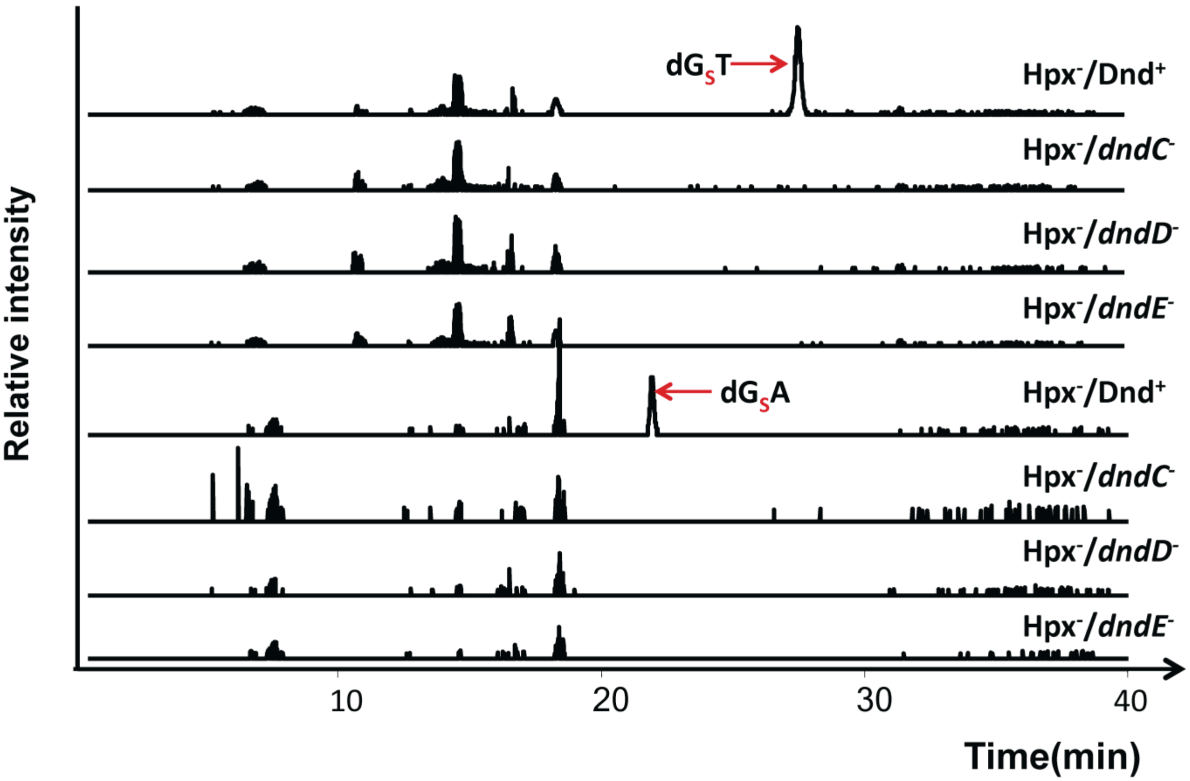

Supplementary Fig. 1.

PT modification detection in the Hpx$^-$/Dnd strains. The Hpx$^-$ mutant was transformed using plasmids containing the *dnd* gene cluster (Dnd$^+$) or the corresponding *dnd* gene in-frame deletion mutants (*dndC*$^-$ - *dndE*$^-$), resulting in *dnd* gene derivatives of Hpx$^-$ (Hpx$^-$/Dnd$^+$, Hpx$^-$/*dndC*$^-$-*dndE*$^-$). PT-linked dinucleotides were detected in the hydrolysate of Hpx$^-$/Dnd$^+$ genome DNA but not in the hydrolysate of Hpx$^-$/Dnd$^-$ genome DNA.

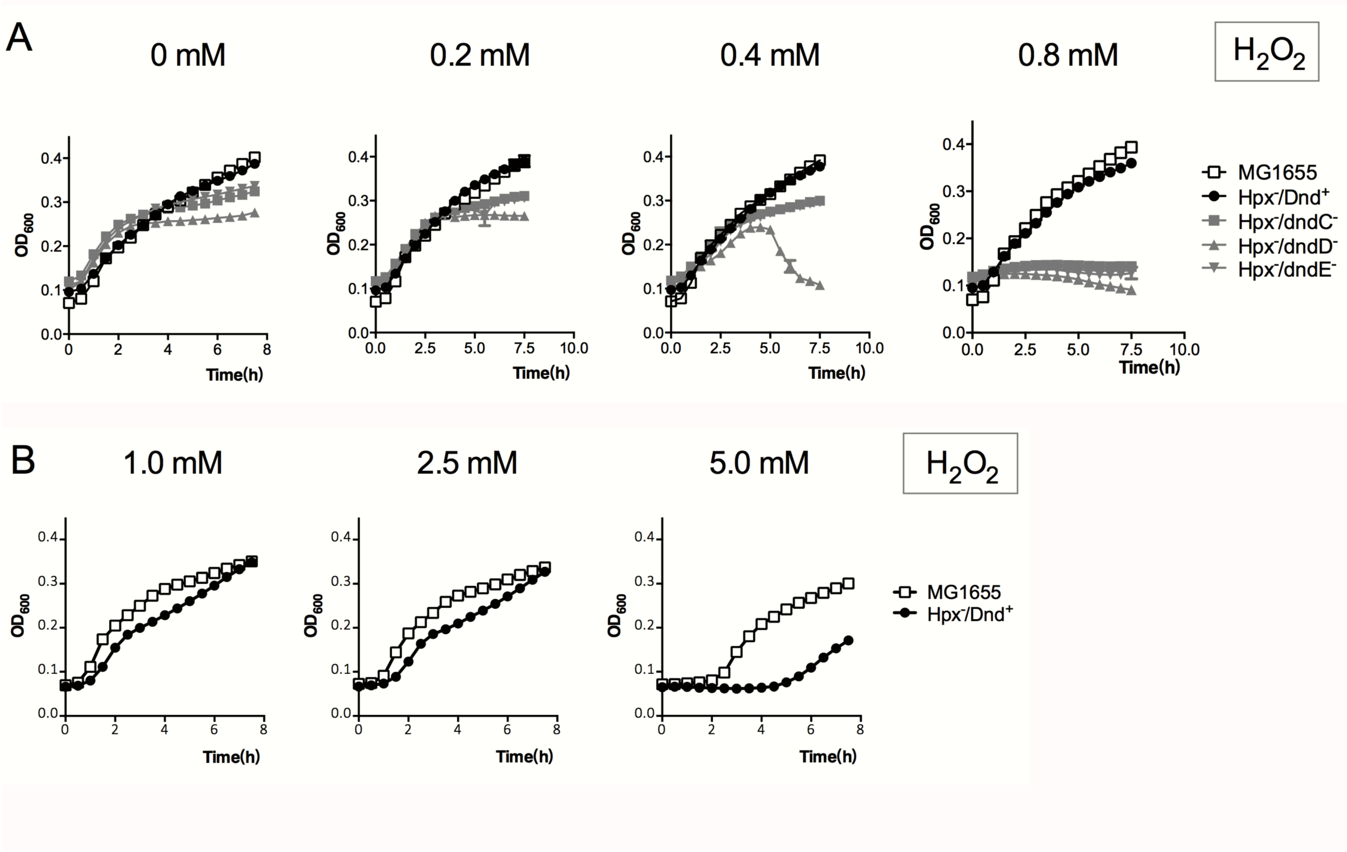

Supplementary Fig. 2

DNA PT modification restores the antioxidation capability of the *E. coli* Hpx^- mutant. (**A**) The cells were treated with 0, 0.2, 0.4, and 0.8 mM H_2O_2, and cell growth was monitored. (**B**) Hpx^-/Dnd^+ and the wild-type *E. coli* strain MG1655 were treated with increasing concentrations of H_2O_2. The growth of the strains was assessed at OD_{600}. The data shown represent the results of three independent experiments, and the error bars indicate the standard deviations.

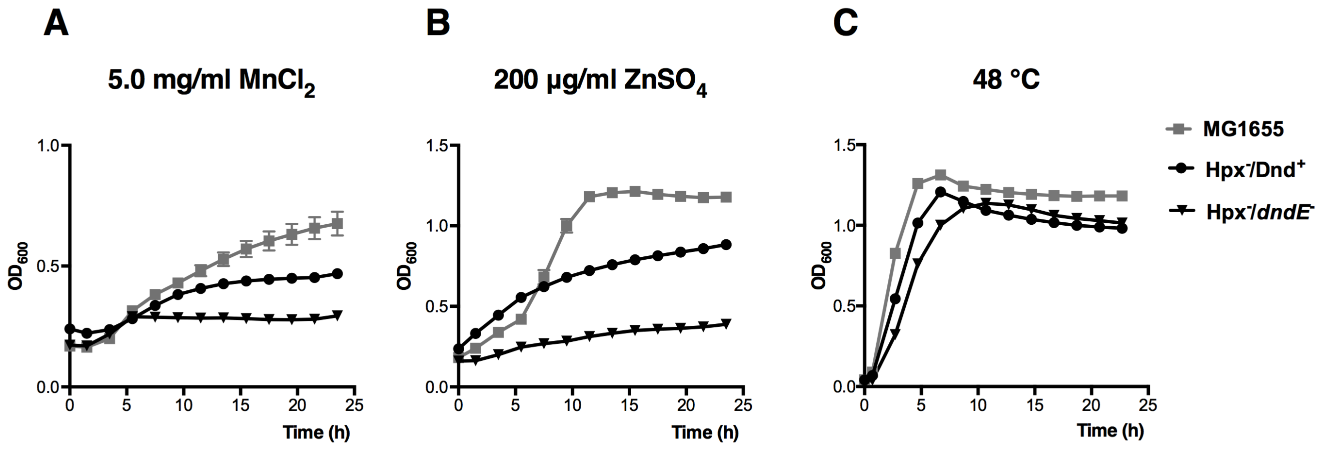

Supplementary Fig. 3

Growth of *E. coli* PT strains under different stress conditions. Hpx$^-$/Dnd$^+$, the wild-type *E. coli* strain MG1655, and Hpx$^-$/*dndE*$^-$ were grown under different $MnCl_2$/$ZnSO_4$/ high temperature stress conditions, and cell growth was monitored. The growth of the strains was detected at OD_{600nm}. The data shown represent the results of three independent experiments, and the error bars indicate the standard deviations.

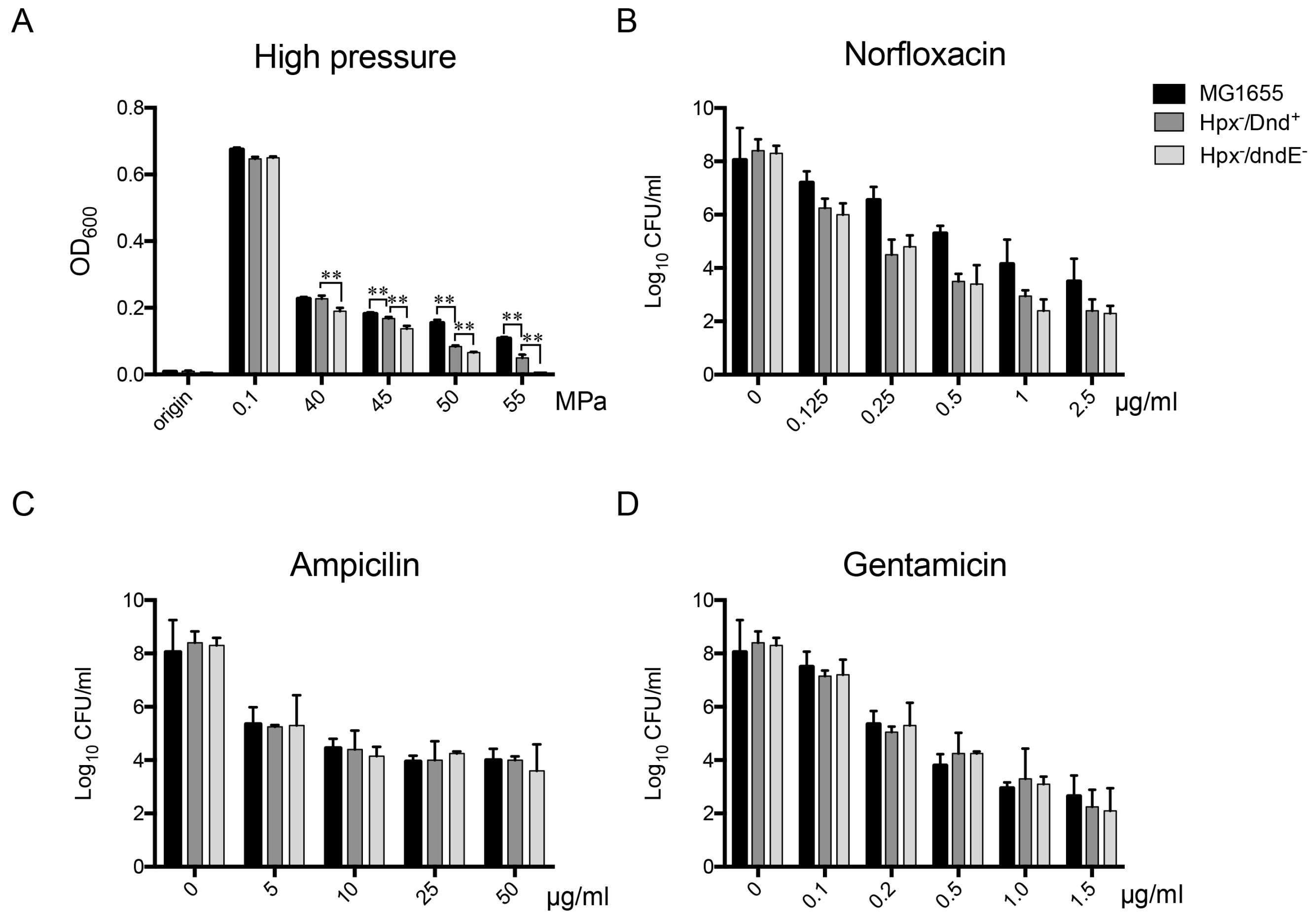

Supplementary Fig. 4

Maximum growth at high pressures and antibiotic killing efficacy of *E. coli* PT strains. (**A**) Hpx$^-$/Dnd$^+$, the wild-type *E. coli* strain MG1655, and Hpx$^-$/*dndE*$^-$ were grown at different high pressures for 48 hours, and the growth of the strains was assessed at OD_{600}. (**B-D**) Colony count after different concentrations of antibiotic treatment. The data shown represent the results of three independent experiments, and the error bars indicate the standard deviations. The data were analyzed by Student's t test. **, P <0.01; ns, not significantly different.

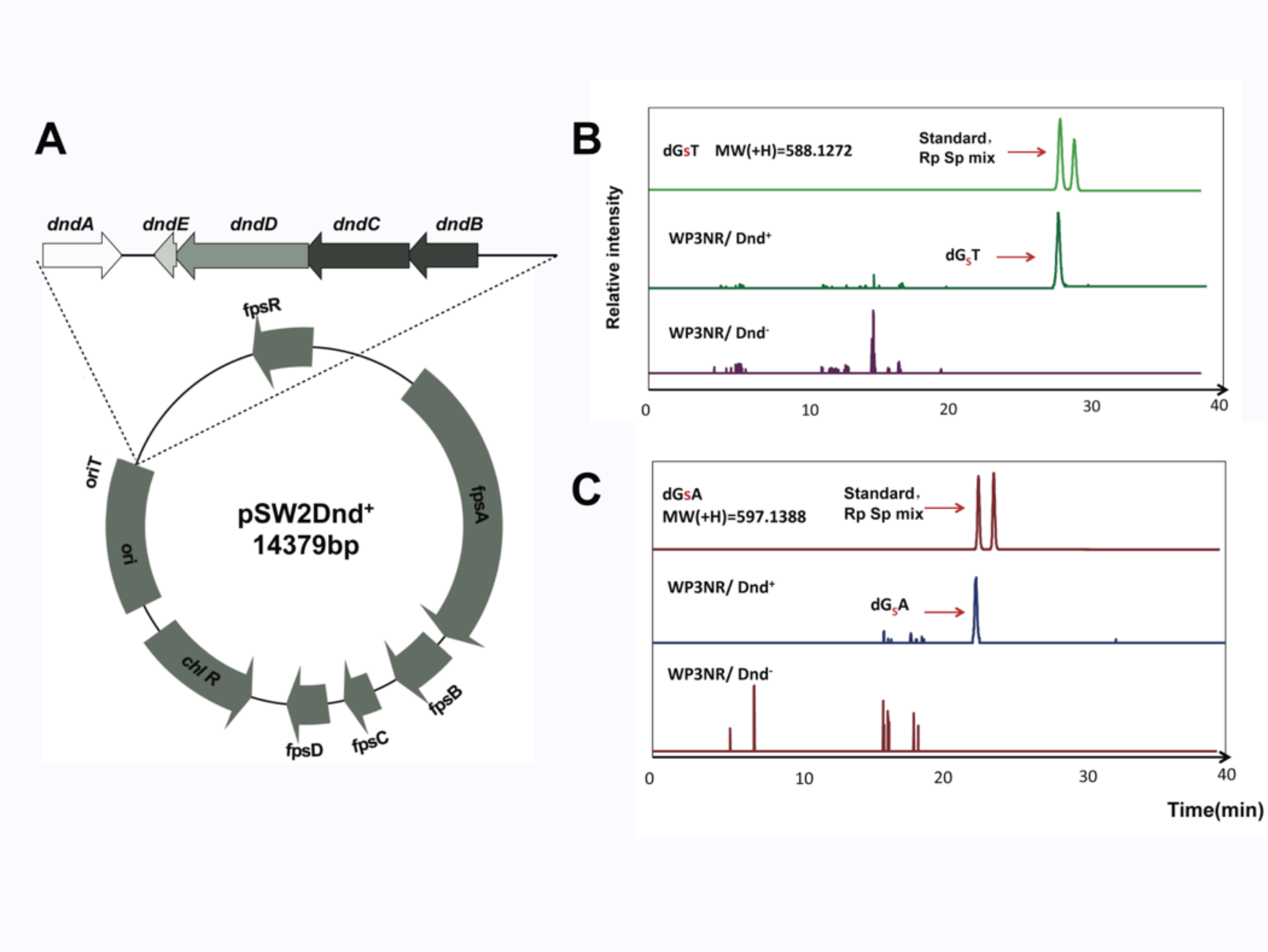

Supplementary Fig. 5

Construction of a DNA PT modification mutant derived from *Shewanella piezotolerans* WP3. (A) Construction of a PT gene cluster clone plasmid functioning in *Shewanella piezotolerans* WP3 strains. *DndA* was cloned from plasmid pJTU3619 and *dndBCDE* genes were cloned from plasmid pJTU1238. (B, C) PT modification detection of WP3NR/ Dnd+. PT-linked dinucleotides were detected in the hydrolysate of WP3NR/ Dnd+ genome DNA but not in the hydrolysate of WP3NR/Dnd- genome DNA.

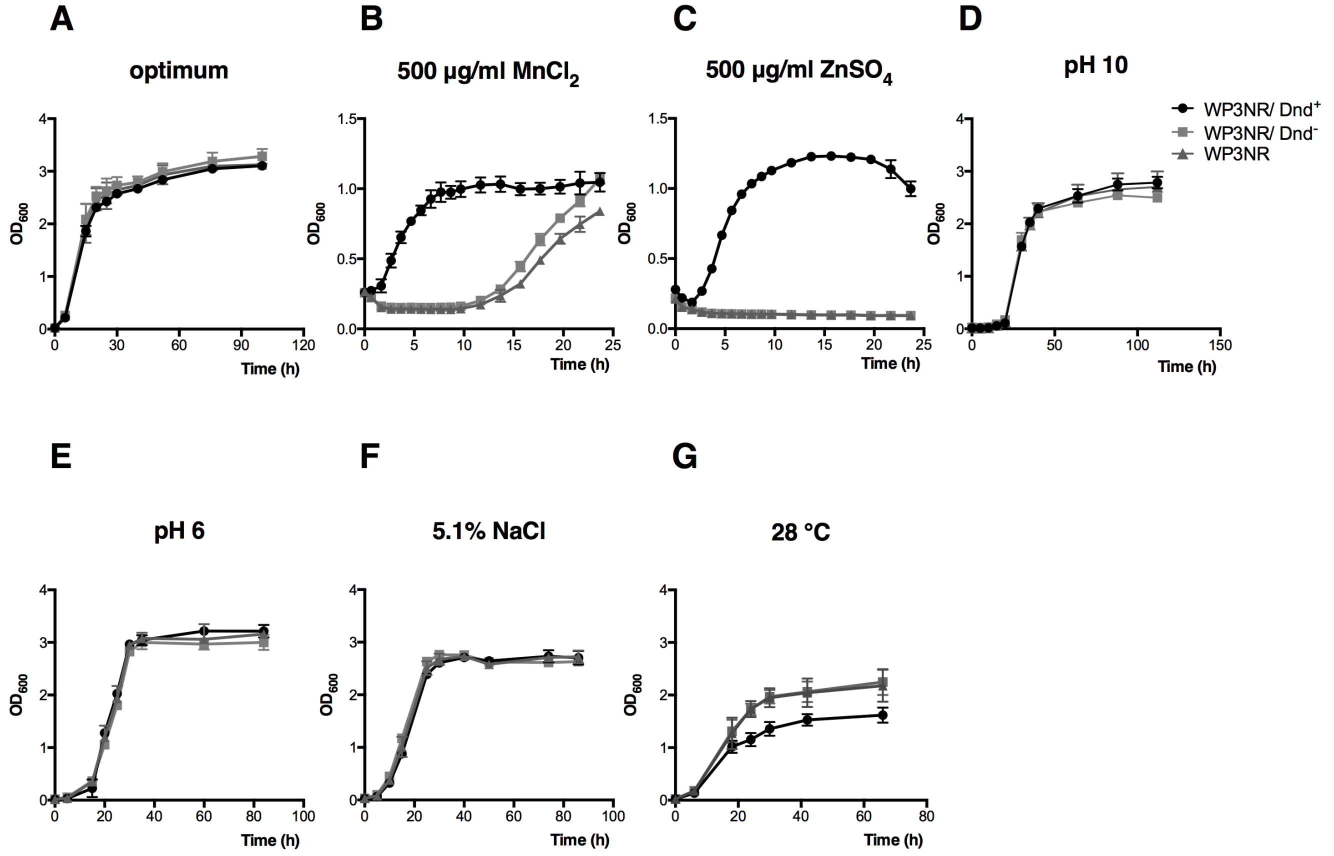

Supplementary Fig. 6

Growth of *Shewanella piezotolerans* PT strains under multiple stresses. WP3NR/Dnd$^+$, WP3NR/Dnd$^-$ and WP3NR were grown under high-temperature, high-salinity, $MnCl_2$, $ZnSO_4$ and pH stress conditions, and the growth of the cells was monitored. The data shown represent the results of three independent experiments, and the error bars indicate the standard deviations.

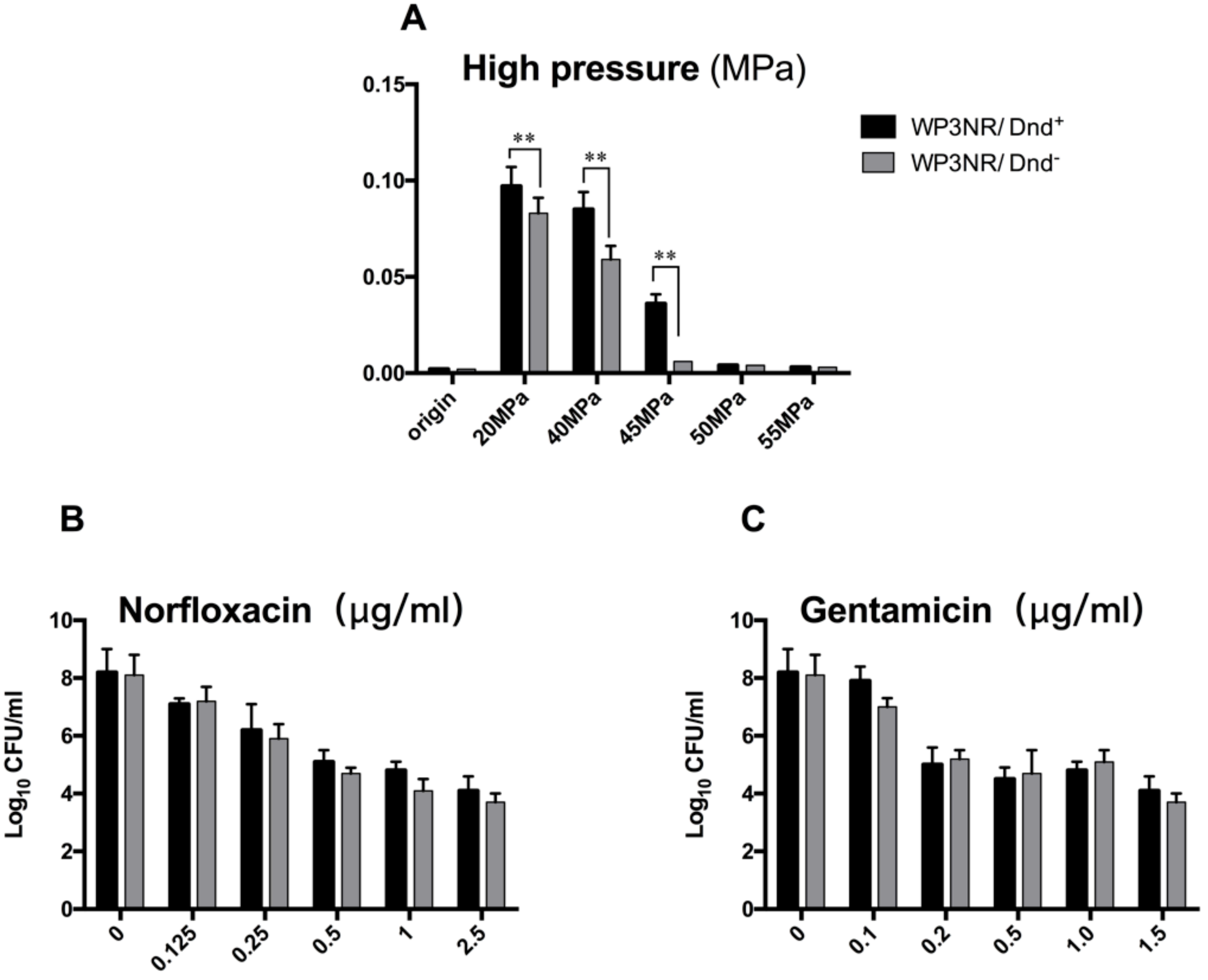

Supplementary Fig. 7

Maximum growth at high pressures and antibiotic killing efficacy of *Shewanella piezotolerans* PT strains. (**A**) WP3NR/Dnd^{+} and WP3NR/Dnd^{-} were grown for 60 hours, and the growth of the strains was assessed at OD_{600}. (**B-C**) Colony count after different concentrations of antibiotic treatment. Ampicillin killing was not detected because WP3 has an ampicillin resistant gene. The data shown represent the results of three independent experiments, and the error bars indicate the standard deviations. The data were analyzed by Student's t test. **, P <0.01.

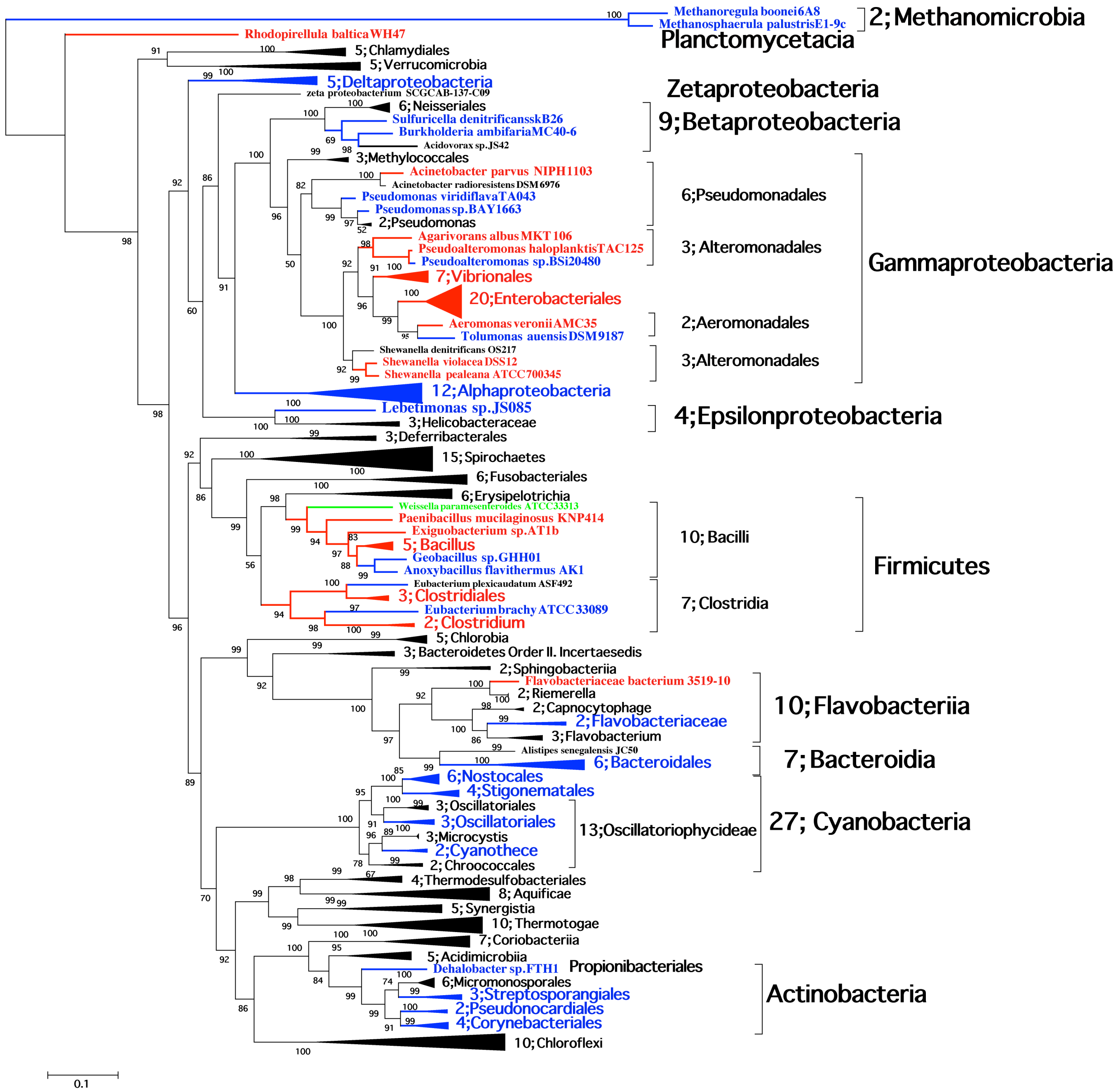

Supplementary Fig. 8

Maximum likelihood-based phylogenetic tree of bacterial 16S rRNA gene sequences, with two Methanomicrobia as the outgroups. Bootstrap values are based on 1000 replicates, and percentages are shown at the nodes. The numbers of genomes in each collapsed clade are displayed before the clade name. Genomes containing both *dndCDE* and *dptFGH* clusters are colored in red, whereas genomes containing only the *dndCDE* cluster are highlighted in blue, and genomes containing only the *dptFGH* cluster are shown in green.

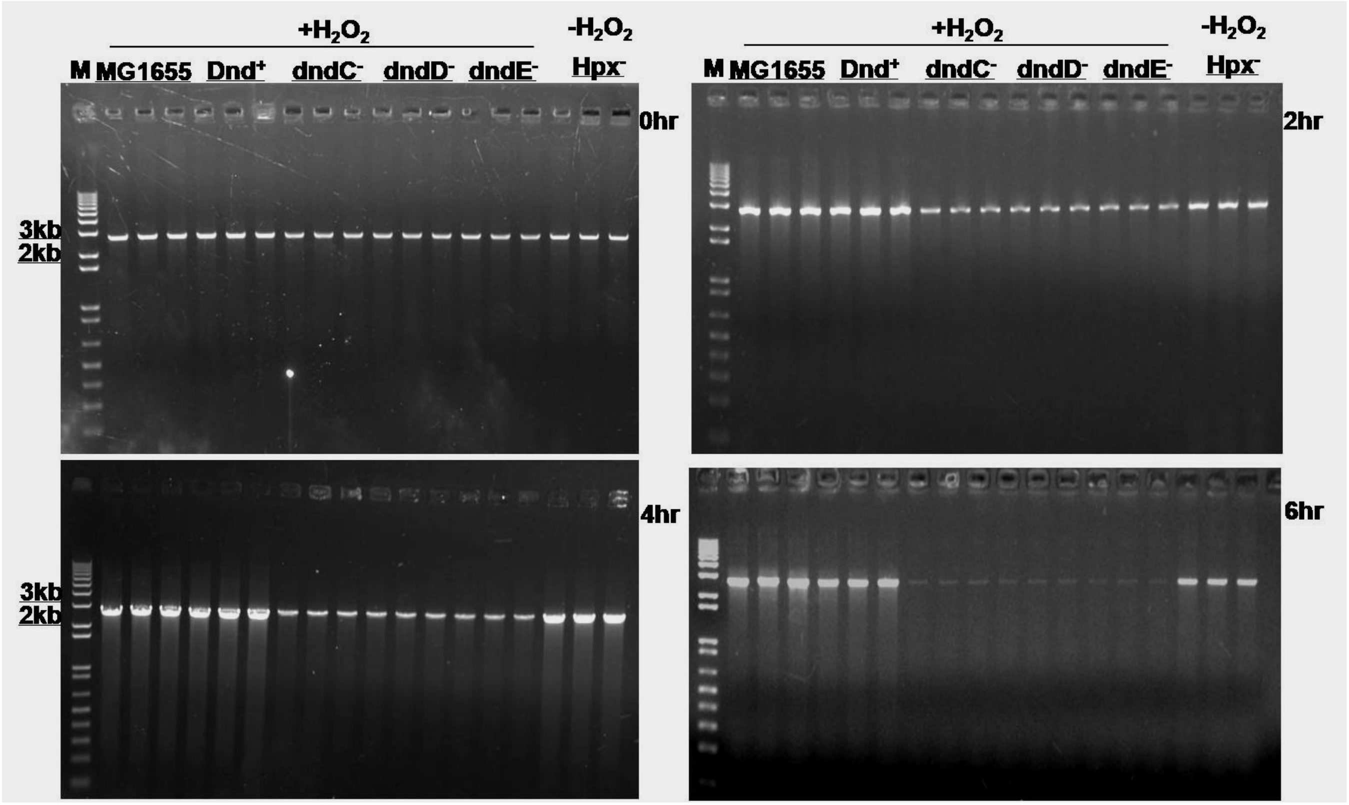

Supplementary Fig. 9

Full-length gels of figure 3.

Supplementary Table 1

Genes involved in antioxidation in the genome of *Shewanella piezotolerance* WP3

locus tag	gene	description
swp_4429	*katE*	Catalase
swp_2307	*soxR*	SOD Transcriptional regulator
swp_3819	*sod*（nickel)	Nickel-containing superoxide dismutase
swp_2754	*sod*（iron)	Superoxide dismutase, Fe
swp_1821	*ahp1*	Alkyl hydroperoxide reductase/ Thiol specific antioxidant/ Mal allergen
swp_2738	*ahp2*	Alkyl hydroperoxide reductase/ Thiol specific antioxidant/ Mal allergen
swp_4205	*ahp3*	Alkyl hydroperoxide reductase/ Thiol specific antioxidant/ Mal allergen
swp_4326<br>swp_4327	*ahp4*	Alkyl hydroperoxide reductase/ Thiol specific antioxidant/ Mal allergen
swp_1272	*gpx*	Glutathione peroxidase
swp_4319	*ohrR*	transcriptional regulator, MarR family
swp_4320	*ohr*	Redox protein, regulator of disulfide bond formation
swp_3799	*oxyR*	KatE,Ahp Transcriptional regulator
swp_2088<br>swp_2866<br>swp_4142<br>swp_4608	*ccp*	Cytochrome-c peroxidase
swp_0167	*dps*	Ferritin and Dps
swp_0860	*bcp*	Bacterioferritin comigratory protein

Supplementary Table 2

Presence of *dnd* and *dpt* gene clusters in publicly available procaryotic genomes in NCBI

Species	Presence of *dnd*/*dpt* gene clusters
Acinetobacter baumannii NIPH 146	Both
Acinetobacter baumannii RUH1486	Both
Acinetobacter parvus NIPH 1103	Both
Acinetobacter sp. YZS-X1-1	Both
Acinetobacter ursingii DSM 16037 = CIP 107286	Both
Aeromonas australiensis CECT 8023	Both
Aeromonas dhakensis CIP 107500	Both
Aeromonas hydrophila 277	Both
Aeromonas jandaei Riv2	Both
Aeromonas sobria CECT 4245	Both
Aeromonas veronii AMC35	Both
Aeromonas veronii ARB3	Both
Agarivorans albus MKT 106	Both
Alteromonas australica H 17	Both
Bacillus aryabhattai GZ03	Both
Bacillus cereus BAG6X1-1	Both
Bacillus cereus E33L	Both
Bacillus cereus Rock1-3	Both
Bacillus cereus VD169	Both
Bacillus firmus DS1	Both
Bacillus isronensis B3W22	Both
Bacillus sp. EB01	Both
Bacillus sp. J33	Both
Bacillus sp. SA2-6	Both

Bacillus sp. SB49	Both
Bacillus thuringiensis serovar pondicheriensis BGSC 4BA1	Both
Bermanella marisrubri RED65	Both
Cedecea neteri ND14a	Both
Citrobacter sp. KTE30	Both
Clostridiales bacterium oral taxon 876 str. F0540	Both
Clostridium bartlettii CAG:1329	Both
Clostridium botulinum 12LNR10-CD	Both
Clostridium botulinum 12LNRI-CD	Both
Clostridium botulinum 29401-CD	Both
Clostridium botulinum 43243-CD	Both
Clostridium botulinum 48212-CD	Both
Clostridium botulinum 50867-CD	Both
Clostridium botulinum 55741-CD	Both
Clostridium botulinum 58272-CD	Both
Clostridium botulinum 58752-CD	Both
Clostridium botulinum 69285-CD	Both
Clostridium botulinum 71840-CD	Both
Clostridium botulinum BKT015925	Both
Clostridium botulinum BKT028387	Both
Clostridium botulinum C/D str. BKT12695	Both
Clostridium botulinum C/D str. Sp77	Both
Clostridium botulinum E3 str. Alaska E43	Both
Clostridium botulinum NCTC 8266	Both
Clostridium botulinum NCTC 8550	Both
Clostridium botulinum V891	Both
Clostridium butyricum DORA_1	Both
Clostridium ljungdahlii DSM 13528	Both

Clostridium perfringens NCTC 8239	Both
Clostridium sp. CAG:127	Both
Clostridium sp. JC272	Both
Cronobacter sakazakii ES35	Both
Desulfitobacterium metallireducens DSM 15288	Both
Desulfovibrio desulfuricans subsp. aestuarii DSM 17919 = ATCC 29578	Both
Dickeya solani M005	Both
Endozoicomonas numazuensis DSM 25634	Both
Enterobacter aerogenes GN02355	Both
Enterobacter aerogenes UCI89	Both
Enterobacter asburiae C1	Both
Enterobacter mori LMG 25706	Both
Enterobacter sp. 638	Both
Enterobacter sp. Bisph1	Both
Enterobacter sp. GN02457	Both
Enterobacter sp. YD4	Both
Enterobacteriaceae bacterium LSJC7	Both
Enterovibrio calviensis 1F-230	Both
Erwinia amylovora 01SFR-BO	Both
Erwinia amylovora ACW56400	Both
Erwinia amylovora ATCC 49946	Both
Erwinia amylovora ATCC BAA-2158	Both
Erwinia amylovora CFBP 1232	Both
Erwinia amylovora CFBP 2585	Both
Erwinia amylovora CFBP1430	Both
Erwinia amylovora Ea266	Both
Erwinia amylovora Ea356	Both
Erwinia amylovora LA635	Both

Erwinia amylovora LA636	Both
Erwinia amylovora LA637	Both
Erwinia amylovora NBRC 12687	Both
Erwinia amylovora UPN527	Both
Escherichia coli 1.2264	Both
Escherichia coli 10.0821	Both
Escherichia coli 113290	Both
Escherichia coli 1-250-04_S1_C1	Both
Escherichia coli 1-250-04_S1_C2	Both
Escherichia coli 1-250-04_S1_C3	Both
Escherichia coli 1365	Both
Escherichia coli 2-052-05_S3_C1	Both
Escherichia coli 2-052-05_S3_C2	Both
Escherichia coli 2-052-05_S3_C3	Both
Escherichia coli 2-156-04_S3_C2	Both
Escherichia coli 2-222-05_S4_C2	Both
Escherichia coli 2-222-05_S4_C3	Both
Escherichia coli 2-474-04_S1_C2	Both
Escherichia coli 2-474-04_S4_C1	Both
Escherichia coli 2-474-04_S4_C2	Both
Escherichia coli 2-474-04_S4_C3	Both
Escherichia coli 3-475-03_S1_C1	Both
Escherichia coli 38.34	Both
Escherichia coli 5-366-08_S3_C1	Both
Escherichia coli 5-366-08_S3_C3	Both
Escherichia coli 53C	Both
Escherichia coli 55989	Both
Escherichia coli 688_ECOL	Both
Escherichia coli 689_ECOL	Both

Escherichia coli 85B	Both
Escherichia coli 8624	Both
Escherichia coli 95.1288	Both
Escherichia coli AI27	Both
Escherichia coli B144-c1	Both
Escherichia coli B7A	Both
Escherichia coli BIDMC 71	Both
Escherichia coli BIDMC 77	Both
Escherichia coli blood-08-1415	Both
Escherichia coli blood-08-1487	Both
Escherichia coli blood-08-1493	Both
Escherichia coli blood-08-1562	Both
Escherichia coli blood-08-1563rep1	Both
Escherichia coli blood-08-1563rep2	Both
Escherichia coli blood-08-1636	Both
Escherichia coli blood-09-1229	Both
Escherichia coli blood-09-1342	Both
Escherichia coli blood-2011-0141	Both
Escherichia coli C496_10	Both
Escherichia coli CFSAN026775	Both
Escherichia coli CFSAN026796	Both
Escherichia coli CFSAN026809	Both
Escherichia coli CFSAN026810	Both
Escherichia coli CVM N33601PS	Both
Escherichia coli CVM N33624PS	Both
Escherichia coli CVM N33720PS	Both
Escherichia coli CVM N34077PS	Both
Escherichia coli CVM N34557PS	Both
Escherichia coli CVM N36093PS	Both

Escherichia coli CVM N36158PS	Both
Escherichia coli CVM N38733PS	Both
Escherichia coli CVM N41498PS	Both
Escherichia coli DEC9A	Both
Escherichia coli E. coli	Both
Escherichia coli E482	Both
Escherichia coli F6700	Both
Escherichia coli FDA506	Both
Escherichia coli H299	Both
Escherichia coli HVH 108 (4-6924867)	Both
Escherichia coli HVH 136 (4-5970458)	Both
Escherichia coli HVH 182 (4-0985554)	Both
Escherichia coli HVH 43 (4-2173468)	Both
Escherichia coli HVH 44 (4-2298570)	Both
Escherichia coli INSLA289	Both
Escherichia coli IS1	Both
Escherichia coli KOEGE 118 (317a)	Both
Escherichia coli KOEGE 7 (16a)	Both
Escherichia coli KTE125	Both
Escherichia coli KTE144	Both
Escherichia coli KTE19	Both
Escherichia coli KTE202	Both
Escherichia coli KTE50	Both
Escherichia coli KTE9	Both
Escherichia coli MGH 57	Both
Escherichia coli MP020940.1	Both
Escherichia coli MS 117-3	Both
Escherichia coli MS 45-1	Both
Escherichia coli O08	Both

Escherichia coli O15:H18 str. K1516	Both
Escherichia coli O174:H21	Both
Escherichia coli OLC-733	Both
Escherichia coli P0304816.1	Both
Escherichia coli P0304816.10	Both
Escherichia coli P0304816.11	Both
Escherichia coli P0304816.12	Both
Escherichia coli P0304816.13	Both
Escherichia coli P0304816.14	Both
Escherichia coli P0304816.15	Both
Escherichia coli P0304816.2	Both
Escherichia coli P0304816.3	Both
Escherichia coli P0304816.4	Both
Escherichia coli P0304816.5	Both
Escherichia coli P0304816.6	Both
Escherichia coli P0304816.7	Both
Escherichia coli P0304816.8	Both
Escherichia coli P0304816.9	Both
Escherichia coli S.93	Both
Escherichia coli SE11	Both
Escherichia coli ST2747	Both
Escherichia coli UMEA 3053-1	Both
Escherichia coli UMEA 3144-1	Both
Escherichia coli UMEA 3176-1	Both
Escherichia coli UMEA 3200-1	Both
Escherichia coli upec-132	Both
Escherichia coli upec-137	Both
Escherichia coli upec-66	Both
Escherichia fergusonii ECD227	Both

Exiguobacterium sp. AB2	Both
Exiguobacterium sp. AT1b	Both
Exiguobacterium sp. S17	Both
Ferrimonas kyonanensis DSM 18153	Both
Flavobacteriaceae bacterium 3519-10	Both
Franconibacter pulveris LMG 24059	Both
Gilliamella apicola SCGC AB-598-B02	Both
Grimontia hollisae CIP 101886	Both
Hahella chejuensis KCTC 2396	Both
Halomonas sp. BC04	Both
Halomonas sp. TD01	Both
Halomonas sp. TG39a	Both
Idiomarina sp. A28L	Both
Klebsiella oxytoca CH2	Both
Klebsiella oxytoca CH5	Both
Klebsiella pneumoniae CICC10011	Both
Lachnospiraceae bacterium 2_1_58FAA	Both
Lachnospiraceae bacterium 6_1_37FAA	Both
Leminorella grimontii ATCC 33999 = DSM 5078	Both
Maribacter forsetii DSM 18668	Both
Marinobacter sp. AK21	Both
Marinobacter sp. C1S70	Both
Marinospirillum insulare DSM 21763	Both
Microscilla marina ATCC 23134	Both
Olleya sp. VCSM12	Both
Paenibacillus dauci H9	Both
Paenibacillus mucilaginosus KNP414	Both
Pantoea ananatis GB1	Both
Paraglaciecola psychrophila 170	Both

Peptoclostridium difficile 70-100-2010	Both
Peptoclostridium difficile CD104	Both
Peptoclostridium difficile CD201	Both
Peptoclostridium difficile CD206	Both
Peptoclostridium difficile CD212	Both
Peptoclostridium difficile CD41	Both
Peptoclostridium difficile E12	Both
Photobacterium angustum ATCC 25915	Both
Photobacterium gaetbulicola Gung47	Both
Photorhabdus luminescens LN2	Both
Photorhabdus temperata subsp. khanii NC19	Both
Pseudoalteromonas haloplanktis TAC125	Both
Pseudoalteromonas luteoviolacea HI1	Both
Pseudoalteromonas piscicida ATCC 15057	Both
Pseudoalteromonas piscicida JCM 20779	Both
Pseudoalteromonas rubra DSM 6842	Both
Pseudoalteromonas ruthenica S3137	Both
Pseudoalteromonas ruthenica S3258	Both
Pseudoalteromonas sp. PAMC 22718	Both
Rheinheimera baltica DSM 14885	Both
Rheinheimera sp. IITR-13	Both
Rhodopirellula baltica WH47	Both
Rouxiella chamberiensis 130333	Both
Ruminococcus gnavus CC55_001C	Both
Salmonella enterica subsp. enterica serovar Bareilly str. CFSAN000183	Both
Salmonella enterica subsp. enterica serovar Bareilly str. CFSAN000184	Both
Salmonella enterica subsp. enterica serovar Bareilly str. CFSAN000187	Both

Salmonella enterica subsp. enterica serovar Bareilly str. CFSAN000192	Both
Salmonella enterica subsp. enterica serovar Bareilly str. CFSAN000194	Both
Salmonella enterica subsp. enterica serovar Bareilly str. CFSAN000195	Both
Salmonella enterica subsp. enterica serovar Bareilly str. CFSAN000196	Both
Salmonella enterica subsp. enterica serovar Bareilly str. CFSAN000198	Both
Salmonella enterica subsp. enterica serovar Bareilly str. CFSAN000199	Both
Salmonella enterica subsp. enterica serovar Bareilly str. CFSAN000201	Both
Salmonella enterica subsp. enterica serovar Bareilly str. CFSAN000202	Both
Salmonella enterica subsp. enterica serovar Bareilly str. CFSAN000203	Both
Salmonella enterica subsp. enterica serovar Bareilly str. CFSAN000204	Both
Salmonella enterica subsp. enterica serovar Bareilly str. CFSAN000206	Both
Salmonella enterica subsp. enterica serovar Bareilly str. CFSAN000208	Both
Salmonella enterica subsp. enterica serovar Bareilly str. CFSAN000209	Both
Salmonella enterica subsp. enterica serovar Bareilly str. CFSAN000210	Both
Salmonella enterica subsp. enterica serovar Bareilly str. CFSAN000214	Both
Salmonella enterica subsp. enterica serovar Bareilly str. CFSAN000216	Both
Salmonella enterica subsp. enterica serovar Bareilly str. CFSAN000218	Both

Salmonella enterica subsp. enterica serovar Bareilly str. CFSAN000219	Both
Salmonella enterica subsp. enterica serovar Bareilly str. CFSAN000220	Both
Salmonella enterica subsp. enterica serovar Bareilly str. CFSAN000222	Both
Salmonella enterica subsp. enterica serovar Bareilly str. CFSAN000223	Both
Salmonella enterica subsp. enterica serovar Bareilly str. CFSAN000224	Both
Salmonella enterica subsp. enterica serovar Bareilly str. CFSAN000225	Both
Salmonella enterica subsp. enterica serovar Bareilly str. CFSAN000227	Both
Salmonella enterica subsp. enterica serovar Bareilly str. CFSAN000233	Both
Salmonella enterica subsp. enterica serovar Cerro FSL R8-0235	Both
Salmonella enterica subsp. enterica serovar Cerro str. 5569	Both
Salmonella enterica subsp. enterica serovar Cerro str. 818	Both
Salmonella enterica subsp. enterica serovar Cerro str. 87	Both
Salmonella enterica subsp. enterica serovar Cerro str. CFSAN001587	Both
Salmonella enterica subsp. enterica serovar Cerro str. CFSAN001588	Both
Salmonella enterica subsp. enterica serovar Cerro str. CFSAN001589	Both
Salmonella enterica subsp. enterica serovar Cerro str. CFSAN001590	Both
Salmonella enterica subsp. enterica serovar Cerro str. CFSAN001669	Both
Salmonella enterica subsp. enterica serovar Cerro str. CFSAN001670	Both

Salmonella enterica subsp. enterica serovar Cerro str. CFSAN001671	Both
Salmonella enterica subsp. enterica serovar Cerro str. CFSAN001673	Both
Salmonella enterica subsp. enterica serovar Cerro str. CFSAN001674	Both
Salmonella enterica subsp. enterica serovar Cerro str. CFSAN001679	Both
Salmonella enterica subsp. enterica serovar Cerro str. CFSAN001680	Both
Salmonella enterica subsp. enterica serovar Cerro str. CFSAN001681	Both
Salmonella enterica subsp. enterica serovar Cerro str. CFSAN001690	Both
Salmonella enterica subsp. enterica serovar Cerro str. CFSAN001691	Both
Salmonella enterica subsp. enterica serovar Cerro str. CFSAN001692	Both
Salmonella enterica subsp. enterica serovar Cerro str. CFSAN001697	Both
Salmonella enterica subsp. enterica serovar Mbandaka str. 2012K-0273	Both
Salmonella enterica subsp. enterica serovar Mbandaka str. CVM N45957	Both
Salmonella enterica subsp. enterica serovar Mbandaka str. CVM N51315	Both
Salmonella enterica subsp. enterica serovar Namur str. 05-2929	Both
Salmonella enterica subsp. enterica serovar Panama str. ATCC 7378	Both
Salmonella enterica subsp. enterica serovar Saintpaul str. 9712	Both
Salmonella enterica subsp. enterica serovar Saintpaul str. CVM N43456	Both

Salmonella enterica subsp. enterica serovar Saintpaul str. CVM N43461	Both
Salmonella enterica subsp. enterica serovar Saintpaul str. CVM N45394	Both
Salmonella enterica subsp. enterica serovar Saintpaul str. CVM N45926	Both
Salmonella enterica subsp. enterica serovar Saintpaul str. CVM N45953	Both
Salmonella enterica subsp. enterica serovar Saintpaul str. CVM N46823	Both
Salmonella enterica subsp. enterica serovar Saintpaul str. CVM N46830	Both
Salmonella enterica subsp. enterica serovar Saintpaul str. CVM N46832	Both
Salmonella enterica subsp. enterica serovar Saintpaul str. CVM N46846	Both
Salmonella enterica subsp. enterica serovar Saintpaul str. CVM N46847	Both
Salmonella enterica subsp. enterica serovar Saintpaul str. CVM N47713	Both
Salmonella enterica subsp. enterica serovar Saintpaul str. CVM N47719	Both
Salmonella enterica subsp. enterica serovar Saintpaul str. CVM N47724	Both
Salmonella enterica subsp. enterica serovar Saintpaul str. CVM N47725	Both
Salmonella enterica subsp. enterica serovar Saintpaul str. CVM N48678	Both
Salmonella enterica subsp. enterica serovar Saintpaul str. CVM N48697	Both
Salmonella enterica subsp. enterica serovar Saintpaul str. CVM N48698	Both
Salmonella enterica subsp. enterica serovar Saintpaul str. CVM N50422	Both

Salmonella enterica subsp. enterica serovar Saintpaul str. CVM N50423	Both
Salmonella enterica subsp. enterica serovar Saintpaul str. CVM N51245	Both
Salmonella enterica subsp. enterica serovar Saintpaul str. CVM N51291	Both
Salmonella enterica subsp. enterica serovar Saintpaul str. JO2008	Both
Salmonella enterica subsp. enterica serovar Saintpaul str. SARA23	Both
Salmonella enterica subsp. enterica serovar Saintpaul str. SARA26	Both
Serratia marcescens FGI94	Both
Serratia plymuthica 4Rx13	Both
Shewanella pealeana ATCC 700345	Both
Shewanella violacea DSS12	Both
Siccibacter turicensis z3032	Both
Sporosarcina ureae DSM 2281	Both
Terrisporobacter glycolicus ATCC 14880 = DSM 1288	Both
Vibrio albensis ATCC 14547	Both
Vibrio albensis VL426	Both
Vibrio campbellii 051011F	Both
Vibrio campbellii CCS02	Both
Vibrio cholerae 133-73	Both
Vibrio cholerae 1587	Both
Vibrio cholerae 2012Env-9	Both
Vibrio cholerae BJG-01	Both
Vibrio cholerae CP1037(10)	Both
Vibrio cholerae MZO-2	Both
Vibrio cholerae MZO-3	Both
Vibrio cholerae ZWU0020	Both

Vibrio crassostreae 9ZC77	Both
Vibrio crassostreae J5-15	Both
Vibrio crassostreae J5-4	Both
Vibrio crassostreae J5-5	Both
Vibrio crassostreae LGP7	Both
Vibrio cyclitrophicus ZF65	Both
Vibrio ezurae NBRC 102218	Both
Vibrio fischeri MJ11	Both
Vibrio genomosp. F10 str. 9ZC157	Both
Vibrio harveyi 823TEZ1	Both
Vibrio harveyi CAIM 1792	Both
Vibrio nigripulchritudo MADA3020	Both
Vibrio nigripulchritudo MADA3021	Both
Vibrio nigripulchritudo MADA3029	Both
Vibrio nigripulchritudo SFn118	Both
Vibrio parahaemolyticus 3259	Both
Vibrio parahaemolyticus B-265	Both
Vibrio parahaemolyticus EKP-008	Both
Vibrio parahaemolyticus K5030	Both
Vibrio parahaemolyticus NIHCB0603	Both
Vibrio parahaemolyticus S001	Both
Vibrio parahaemolyticus S148	Both
Vibrio parahaemolyticus S152	Both
Vibrio parahaemolyticus S160	Both
Vibrio parahaemolyticus S164	Both
Vibrio parahaemolyticus S166	Both
Vibrio Parahaemolyticus VIP4-0439	Both
Vibrio parahaemolyticus VIP4-0445	Both
Vibrio sp. JCM 18905	Both

Vibrio sp. JCM 19232	Both
Vibrio splendidus ZS-139	Both
Vibrio tasmaniensis 1F-267	Both
Vibrio vulnificus 491771	Both
Vibrio vulnificus ATCC 33147	Both
Vibrio vulnificus B2	Both
Vibrio vulnificus BAA87	Both
Vibrio vulnificus NBRC 15645 = ATCC 27562	Both
Vibrio vulnificus NV22	Both
Vibrio vulnificus VV4-03	Both
Vibrio vulnificus VV9-09	Both
Yersinia enterocolitica FE80217	Both
Yersinia intermedia FCF130	Both
Yersinia intermedia FCF84	Both
Yersinia ruckeri YRB	Both
Clostridium asparagiforme DSM 15981	*dnd*
Clostridium clostridioforme CM201	*dnd*
Clostridium papyrosolvens DSM 2782	*dnd*
Oscillatoria sp. PCC 6506	*dnd*
Scytonema hofmanni UTEX 2349	*dnd*
Acaryochloris sp. CCMEE 5410	*dnd*
Acetivibrio cellulolyticus CD2	*dnd*
Acetobacter aceti NBRC 14818	*dnd*
Acidobacterium capsulatum ATCC 51196	*dnd*
Acinetobacter guillouiae CIP 63.46	*dnd*
Acinetobacter guillouiae KCTC 23200	*dnd*
Acinetobacter guillouiae MSP4-18	*dnd*
Actinomadura atramentaria DSM 43919	*dnd*
Agrobacterium sp. 10MFCol1.1	*dnd*

alpha proteobacterium HIMB5	*dnd*
Amycolatopsis halophila YIM 93223	*dnd*
Amycolatopsis japonica MG417-CF17	*dnd*
Amycolatopsis jejuensis NRRL B-24427	*dnd*
Amycolatopsis orientalis DSM 43388	*dnd*
Anabaena cylindrica PCC 7122	*dnd*
Anabaena sp. 90	*dnd*
Anabaena sp. PCC 7108	*dnd*
Anabaena variabilis ATCC 29413	*dnd*
Aneurinibacillus migulanus nagano	*dnd*
Anoxybacillus flavithermus AK1	*dnd*
Anoxybacillus flavithermus subsp. yunnanensis str. E13	*dnd*
Anoxybacillus sp. KU2-6(11)	*dnd*
Aphanizomenon flos-aquae NIES-81	*dnd*
Aphanocapsa montana BDHKU210001	*dnd*
Arthrospira sp. TJSD091	*dnd*
Asticcacaulis sp. AC466	*dnd*
Bacillus cereus SDA KA 96	*dnd*
Bacillus okhensis Kh10-101	*dnd*
Bacillus thuringiensis HD1011	*dnd*
Bacteroides faecis MAJ27	*dnd*
Bacteroides finegoldii CL09T03C10	*dnd*
Bacteroides fragilis str. 2-F-2 #4	*dnd*
Bacteroides fragilis str. 2-F-2 #5	*dnd*
Bacteroides fragilis str. 2-F-2 #7	*dnd*
Bacteroides sp. 2_1_33B	*dnd*
Bacteroides vulgatus dnLKV7	*dnd*
Bacteroides xylanisolvens XB1A	*dnd*
Bacteroidetes bacterium JGI 0000119-L22	*dnd*

Beggiatoa sp. PS	*dnd*
Belliella baltica DSM 15883	*dnd*
Burkholderia ambifaria MC40-6	*dnd*
Burkholderia contaminans FFH2055	*dnd*
Burkholderia pseudomallei 12-40	*dnd*
Burkholderia pseudomallei 15-10	*dnd*
Burkholderia pseudomallei 153	*dnd*
Burkholderia pseudomallei 15-40	*dnd*
Burkholderia pseudomallei 17	*dnd*
Burkholderia pseudomallei 33	*dnd*
Burkholderia pseudomallei 4033-10	*dnd*
Burkholderia pseudomallei 4044-138	*dnd*
Burkholderia pseudomallei 4094-0081	*dnd*
Burkholderia pseudomallei 48	*dnd*
Burkholderia pseudomallei 54	*dnd*
Burkholderia pseudomallei AH1	*dnd*
Burkholderia pseudomallei Bp22	*dnd*
Burkholderia pseudomallei D7 3230-3018	*dnd*
Burkholderia pseudomallei DR08726/01	*dnd*
Burkholderia pseudomallei I6 4043-3096	*dnd*
Burkholderia pseudomallei MSHR1029	*dnd*
Burkholderia pseudomallei MSHR1328	*dnd*
Burkholderia pseudomallei MSHR1357	*dnd*
Burkholderia pseudomallei MSHR4378	*dnd*
Burkholderia pseudomallei MSHR5569	*dnd*
Burkholderia pseudomallei QCMRI_BP18	*dnd*
Burkholderia pseudomallei SW1	*dnd*
Burkholderia pseudomallei SW9	*dnd*
Burkholderia pseudomallei TSV 48	*dnd*

Burkholderiaceae bacterium 16	*dnd*
Butyrivibrio sp. AE3006	*dnd*
Calothrix sp. PCC 7103	*dnd*
Calothrix sp. PCC 7507	*dnd*
Candidatus Magnetoovum chiemensis CS-04	*dnd*
Candidatus Nitrosopumilus salaria BD31	*dnd*
Candidatus Pelagibacter ubique HTCC1002	*dnd*
Candidatus Schmidhempelia bombi str. Bimp	*dnd*
Candidatus Thiomargarita nelsonii	*dnd*
Caulobacter sp. AP07	*dnd*
Chelativorans sp. BNC1	*dnd*
Chromobacterium haemolyticum T124	*dnd*
Chromobacterium subtsugae F49	*dnd*
Chroococcales cyanobacterium CENA595	*dnd*
Chroococcidiopsis thermalis PCC 7203	*dnd*
Chryseobacterium sp. OV259	*dnd*
Citrobacter koseri ATCC BAA-895	*dnd*
Clostridiaceae bacterium MS3	*dnd*
Clostridiales bacterium VE202-06	*dnd*
Clostridium beijerinckii 59B	*dnd*
Clostridium beijerinckii NCIMB 14988	*dnd*
Clostridium novyi A str. 4552	*dnd*
Clostridium novyi A str. GD211209	*dnd*
Clostridium sporogenes CDC23284	*dnd*
Coleofasciculus chthonoplastes PCC 7420	*dnd*
Comamonas testosteroni D4	*dnd*
Crinalium epipsammum PCC 9333	*dnd*
Crocosphaera watsonii WH 0003	*dnd*
Crocosphaera watsonii WH 0005	*dnd*

Crocosphaera watsonii WH 0401	*dnd*
Crocosphaera watsonii WH 0402	*dnd*
Crocosphaera watsonii WH 8501	*dnd*
cyanobacterium PCC 7702	*dnd*
Cyanothece sp. ATCC 51142	*dnd*
Cyanothece sp. ATCC 51472	*dnd*
Cyanothece sp. CCY0110	*dnd*
Cyanothece sp. PCC 7424	*dnd*
Cyanothece sp. PCC 7822	*dnd*
Cyanothece sp. PCC 8801	*dnd*
Cyanothece sp. PCC 8802	*dnd*
Cyclobacteriaceae bacterium AK24	*dnd*
Cylindrospermopsis raciborskii CS-505	*dnd*
Cylindrospermum stagnale PCC 7417	*dnd*
Dehalobacter sp. FTH1	*dnd*
Desulfatibacillum alkenivorans AK-01	*dnd*
Desulfatirhabdium butyrativorans DSM 18734	*dnd*
Desulfonatronum thioautotrophicum ASO4-1	*dnd*
Desulfospira joergensenii DSM 10085	*dnd*
Desulfovibrio africanus str. Walvis Bay	*dnd*
Desulfovibrio cf. magneticus IFRC170	*dnd*
Desulfovibrio inopinatus DSM 10711	*dnd*
Desulfovibrio oxyclinae DSM 11498	*dnd*
Dickeya dadantii 3937	*dnd*
Dolichospermum circinale AWQC131C	*dnd*
Dolichospermum circinale AWQC310F	*dnd*
Enterobacter asburiae LF7a	*dnd*
Enterobacter cloacae FDA_MicroDB_70	*dnd*
Enterobacter cloacae FDAARGOS_68	*dnd*

Enterobacter cloacae GN02767	*dnd*
Enterobacter cloacae subsp. cloacae NCTC 9394	*dnd*
Enterobacter cloacae subsp. cloacae WCHECl-1060	*dnd*
Enterococcus gallinarum EGD-AAK12	*dnd*
Escherichia coli PCN061	*dnd*
Escherichia coli upec-284	*dnd*
Eubacterium brachy ATCC 33089	*dnd*
Eubacterium plexicaudatum ASF492	*dnd*
Eubacterium ramulus ATCC 29099	*dnd*
Firmicutes bacterium ASF500	*dnd*
Firmicutes bacterium CAG:270	*dnd*
Fischerella muscicola SAG 1427-1 = PCC 73103	*dnd*
Fischerella sp. JSC-11	*dnd*
Fischerella sp. PCC 9339	*dnd*
Fischerella sp. PCC 9431	*dnd*
Fischerella sp. PCC 9605	*dnd*
Fischerella thermalis PCC 7521	*dnd*
Flavobacterium indicum GPTSA100-9 = DSM 17447	*dnd*
Flavobacterium sp. 316	*dnd*
Flexibacter litoralis DSM 6794	*dnd*
gamma proteobacterium NOR5-3	*dnd*
gamma proteobacterium WG36	*dnd*
Geminocystis sp. NIES-3708	*dnd*
Geminocystis sp. NIES-3709	*dnd*
Geobacillus kaustophilus Et2/3	*dnd*
Geobacillus sp. GHH01	*dnd*
Geobacillus stearothermophilus NUB3621	*dnd*
Geobacter bremensis R1	*dnd*
Geobacter uraniireducens Rf4	*dnd*

Gloeocapsa sp. PCC 7428	*dnd*
Gordonia sputi NBRC 100414	*dnd*
Hassallia byssoidea VB512170	*dnd*
Hyphomonas oceanitis SCH89	*dnd*
Idiomarina sp. 28-8	*dnd*
Jiangella alkaliphila KCTC 19222	*dnd*
Kamptonema formosum PCC 6407	*dnd*
Kandleria vitulina WCE2011	*dnd*
Klebsiella oxytoca CH2	*dnd*
Kordia algicida OT-1	*dnd*
Lachnospiraceae bacterium AC2012	*dnd*
Lachnospiraceae bacterium FE2018	*dnd*
Lachnospiraceae bacterium oral taxon 082 str. F0431	*dnd*
Lebetimonas sp. JH369	*dnd*
Lebetimonas sp. JS085	*dnd*
Lebetimonas sp. JS170	*dnd*
Leptolyngbya sp. JSC-1	*dnd*
Leptolyngbya sp. KIOST-1	*dnd*
Leptolyngbya sp. PCC 6406	*dnd*
Limnoraphis robusta CS-951	*dnd*
Marinobacter sp. EN3	*dnd*
Mastigocladopsis repens PCC 10914	*dnd*
Mastigocladus laminosus UU774	*dnd*
Mesonia mobilis DSM 19841	*dnd*
Mesorhizobium amorphae CCNWGS0123	*dnd*
Mesorhizobium sp. WSM3224	*dnd*
Methanoregula boonei 6A8	*dnd*
Methanosarcina mazei 1.H.A.1A.1	*dnd*
Methanosarcina mazei 1.H.A.1A.3	*dnd*

Methanosarcina mazei 1.H.A.1A.6	*dnd*
Methanosarcina mazei 1.H.A.2.3	*dnd*
Methanosarcina mazei 1.H.A.2.7	*dnd*
Methanosarcina mazei 1.H.A.2.8	*dnd*
Methanosarcina mazei 1.H.M.0.1	*dnd*
Methanosarcina mazei 1.H.M.1A.1	*dnd*
Methanosarcina mazei 1.H.M.1A.2	*dnd*
Methanosarcina mazei 1.H.M.1A.3	*dnd*
Methanosarcina mazei 1.H.M.2.2	*dnd*
Methanosarcina mazei 1.H.M.2.3	*dnd*
Methanosarcina mazei 1.H.M.2.4	*dnd*
Methanosarcina mazei 1.H.T.2.1	*dnd*
Methanosarcina mazei 1.H.T.2.3	*dnd*
Methanosarcina mazei 1.H.T.2.5	*dnd*
Methanosarcina mazei 2.F.A.2.3	*dnd*
Methanosarcina mazei 3.F.A.1A.1	*dnd*
Methanosarcina mazei 3.F.A.1B.1	*dnd*
Methanosphaerula palustris E1-9c	*dnd*
Microchaete sp. PCC 7126	*dnd*
Microcoleus sp. PCC 7113	*dnd*
Microcoleus vaginatus FGP-2	*dnd*
Moorea producens 3L	*dnd*
Moorella glycerini NMP	*dnd*
Mycobacterium abscessus 1948	*dnd*
Mycobacterium abscessus 3A-0119-R	*dnd*
Mycobacterium abscessus 3A-0122-*R*	*dnd*
Mycobacterium abscessus 3A-0122-S	*dnd*
Mycobacterium abscessus 3A-0731	*dnd*
Mycobacterium abscessus 3A-0810-R	*dnd*

Mycobacterium abscessus 3A-0930-R	*dnd*
Mycobacterium abscessus 3A-0930-S	*dnd*
Mycobacterium abscessus 6G-0125-R	*dnd*
Mycobacterium abscessus 6G-0125-S	*dnd*
Mycobacterium abscessus 6G-0212	*dnd*
Mycobacterium abscessus 6G-0728-R	*dnd*
Mycobacterium abscessus 6G-0728-S	*dnd*
Mycobacterium abscessus 6G-1108	*dnd*
Mycobacterium abscessus ATCC 19977	*dnd*
Mycobacterium abscessus MAB_110811_2726	*dnd*
Mycobacterium abscessus PAP001	*dnd*
Mycobacterium abscessus PAP002	*dnd*
Mycobacterium abscessus PAP004	*dnd*
Mycobacterium abscessus PAP020	*dnd*
Mycobacterium abscessus PAP077	*dnd*
Mycobacterium abscessus subsp. bolletii 103	*dnd*
Mycobacterium abscessus subsp. bolletii MA 1948	*dnd*
Mycobacterium abscessus subsp. bolletii MC1518	*dnd*
Mycobacterium abscessus UC95	*dnd*
Mycobacterium abscessus V06705	*dnd*
Mycobacterium chelonae 1518	*dnd*
Mycobacterium fortuitum Z58	*dnd*
Mycobacterium kansasii Z61	*dnd*
Mycobacterium parascrofulaceum ATCC BAA-614	*dnd*
Mycobacterium phlei RIVM601174	*dnd*
Mycobacterium vaccae ATCC 25954	*dnd*
Natronorubrum bangense JCM 10635	*dnd*
Nocardia brasiliensis ATCC 700358	*dnd*
Nocardiopsis sp. CNS639	*dnd*

Nocardiopsis sp. CNT312	*dnd*
Nocardiopsis synnemataformans DSM 44143	*dnd*
Nodosilinea nodulosa PCC 7104	*dnd*
Nodularia spumigena CCY9414	*dnd*
Nostoc punctiforme PCC 73102	*dnd*
Nostoc sp. PCC 7120	*dnd*
Oleispira antarctica RB-8	*dnd*
Oscillatoria acuminata PCC 6304	*dnd*
Oscillatoria nigro-viridis PCC 7112	*dnd*
Oscillatoria sp. PCC 10802	*dnd*
Oscillospiraceae bacterium VE202-24	*dnd*
Parabacteroides goldsteinii CL02T12C30	*dnd*
Parabacteroides goldsteinii DSM 19448 = WAL 12034	*dnd*
Paraprevotella xylaniphila YIT 11841	*dnd*
Pectobacterium carotovorum subsp. Brasiliense YC D52	*dnd*
Peptoclostridium difficile 342	*dnd*
Peptoclostridium difficile 6041	*dnd*
Peptoclostridium difficile ATCC 9689 = DSM 1296	*dnd*
Peptoclostridium difficile CD129	*dnd*
Peptoclostridium difficile CD131	*dnd*
Peptoclostridium difficile CD149	*dnd*
Peptoclostridium difficile CD166	*dnd*
Peptoclostridium difficile CD181	*dnd*
Peptoclostridium difficile CD200	*dnd*
Peptoclostridium difficile CD22	*dnd*
Peptoclostridium difficile CD40	*dnd*
Peptoclostridium difficile CD43	*dnd*
Peptoclostridium difficile CD47	*dnd*
Peptoclostridium difficile CD86	*dnd*

Peptoclostridium difficile CD92	*dnd*
Peptoclostridium difficile DA00128	*dnd*
Peptoclostridium difficile DA00167	*dnd*
Peptoclostridium difficile DA00238	*dnd*
Peptoclostridium difficile DA00273	*dnd*
Peptoclostridium difficile DA00305	*dnd*
Peptoclostridium difficile DA00307	*dnd*
Peptoclostridium difficile E23	*dnd*
Peptoclostridium difficile F152	*dnd*
Peptoclostridium difficile P1	*dnd*
Peptoclostridium difficile P6	*dnd*
Peptoclostridium difficile P69	*dnd*
Peptoclostridium difficile P8	*dnd*
Peptoclostridium difficile QCD-63q42	*dnd*
Peptoclostridium difficile T10	*dnd*
Peptoclostridium difficile T14	*dnd*
Peptococcaceae bacterium BRH_c4a	*dnd*
Planktothrix agardhii NIVA-CYA 126/8	*dnd*
Planktothrix agardhii NIVA-CYA 15	*dnd*
Planktothrix agardhii NIVA-CYA 34	*dnd*
Planktothrix agardhii NIVA-CYA 56/3	*dnd*
Planktothrix mougeotii NIVA-CYA 405	*dnd*
Planktothrix prolifica NIVA-CYA 406	*dnd*
Planktothrix prolifica NIVA-CYA 540	*dnd*
Planktothrix prolifica NIVA-CYA 98	*dnd*
Planktothrix rubescens NIVA-CYA 407	*dnd*
Polaribacter sp. Hel_I_88	*dnd*
Porphyrobacter sp. HL-46	*dnd*
Porphyromonadaceae bacterium ING2-E5B	*dnd*

Prevotella amnii CRIS 21A-A	*dnd*
Prevotella bivia DSM 20514	*dnd*
Prevotella histicola JCM 15637 = DNF00424	*dnd*
Proteus mirabilis PR03	*dnd*
Pseudanabaena biceps PCC 7429	*dnd*
Pseudoalteromonas sp. BSi20480	*dnd*
Pseudomonas aeruginosa DK2	*dnd*
Pseudomonas fluorescens Pf0-1	*dnd*
Pseudomonas sp. BAY1663	*dnd*
Pseudomonas sp. GM16	*dnd*
Pseudomonas sp. GM24	*dnd*
Pseudomonas stutzeri BAL361	*dnd*
Pseudomonas viridiflava TA043	*dnd*
Raphidiopsis brookii D9	*dnd*
Rhizobium etli CNPAF512	*dnd*
Rhizobium larrymoorei ATCC 51759	*dnd*
Rhizobium leguminosarum bv. trifolii WSM1689	*dnd*
Rhizobium mesoamericanum STM6155	*dnd*
Rhodococcus fascians A78	*dnd*
Riemerella anatipestifer 153	*dnd*
Riemerella anatipestifer ATCC 11845 = DSM 15868	*dnd*
Riemerella anatipestifer RA-CH-2	*dnd*
Riemerella anatipestifer RA-SG	*dnd*
Riemerella anatipestifer RA-YM	*dnd*
Riemerella anatipestifer Yb2	*dnd*
Rivularia sp. PCC 7116	*dnd*
Roseburia inulinivorans CAG:15	*dnd*
Roseobacter denitrificans OCh 114	*dnd*
Ruminococcus albus SY3	*dnd*

Ruminococcus flavefaciens 17	*dnd*
Salinispora pacifica CNR894	*dnd*
Salinispora pacifica CNY330	*dnd*
Salmonella enterica subsp. diarizonae serovar 60:r:e,n,x,z15 str. 01-0170	*dnd*
Salmonella enterica subsp. enterica serovar I 8,20:-:z6 CVM N43471	*dnd*
Salmonella enterica subsp. enterica serovar Infantis str. SARB27	*dnd*
Salmonella enterica subsp. enterica serovar Kentucky str. 0253	*dnd*
Salmonella enterica subsp. enterica serovar Kentucky str. 13562	*dnd*
Salmonella enterica subsp. enterica serovar Kentucky str. 20793	*dnd*
Salmonella enterica subsp. enterica serovar Kentucky str. 22694	*dnd*
Salmonella enterica subsp. enterica serovar Kentucky str. 29166	*dnd*
Salmonella enterica subsp. enterica serovar Kentucky str. 29439	*dnd*
Salmonella enterica subsp. enterica serovar Kentucky str. 5349	*dnd*
Salmonella enterica subsp. enterica serovar Kentucky str. ABB07-SB3057-2	*dnd*
Salmonella enterica subsp. enterica serovar Kentucky str. ABB1087-1	*dnd*
Salmonella enterica subsp. enterica serovar Kentucky str. ABBSB1008-2	*dnd*
Salmonella enterica subsp. enterica serovar Kentucky str. CDC 191	*dnd*
Salmonella enterica subsp. enterica serovar Kentucky str. CVM N43447	*dnd*
Salmonella enterica subsp. enterica serovar Kentucky str. CVM N43448	*dnd*

Salmonella enterica subsp. enterica serovar Kentucky str. CVM N43450	*dnd*
Salmonella enterica subsp. enterica serovar Kentucky str. CVM N43455	*dnd*
Salmonella enterica subsp. enterica serovar Kentucky str. CVM N43465	*dnd*
Salmonella enterica subsp. enterica serovar Kentucky str. CVM N43466	*dnd*
Salmonella enterica subsp. enterica serovar Kentucky str. CVM N43478	*dnd*
Salmonella enterica subsp. enterica serovar Kentucky str. CVM N43820	*dnd*
Salmonella enterica subsp. enterica serovar Kentucky str. CVM N43835	*dnd*
Salmonella enterica subsp. enterica serovar Kentucky str. CVM N44708	*dnd*
Salmonella enterica subsp. enterica serovar Kentucky str. CVM N45412	*dnd*
Salmonella enterica subsp. enterica serovar Kentucky str. CVM N45934	*dnd*
Salmonella enterica subsp. enterica serovar Kentucky str. CVM N45937	*dnd*
Salmonella enterica subsp. enterica serovar Kentucky str. CVM N45939	*dnd*
Salmonella enterica subsp. enterica serovar Kentucky str. CVM N45944	*dnd*
Salmonella enterica subsp. enterica serovar Kentucky str. CVM N46820	*dnd*
Salmonella enterica subsp. enterica serovar Kentucky str. CVM N46849	*dnd*
Salmonella enterica subsp. enterica serovar Kentucky str. CVM N46857	*dnd*
Salmonella enterica subsp. enterica serovar Kentucky str. CVM N47718	*dnd*

Salmonella enterica subsp. enterica serovar Kentucky str. CVM N47720	*dnd*
Salmonella enterica subsp. enterica serovar Kentucky str. CVM N47721	*dnd*
Salmonella enterica subsp. enterica serovar Kentucky str. CVM N47723	*dnd*
Salmonella enterica subsp. enterica serovar Kentucky str. CVM N47729	*dnd*
Salmonella enterica subsp. enterica serovar Kentucky str. CVM N47730	*dnd*
Salmonella enterica subsp. enterica serovar Kentucky str. CVM N48688	*dnd*
Salmonella enterica subsp. enterica serovar Kentucky str. CVM N48707	*dnd*
Salmonella enterica subsp. enterica serovar Kentucky str. CVM N48710	*dnd*
Salmonella enterica subsp. enterica serovar Kentucky str. CVM N48711	*dnd*
Salmonella enterica subsp. enterica serovar Kentucky str. CVM N50419	*dnd*
Salmonella enterica subsp. enterica serovar Kentucky str. CVM N50421	*dnd*
Salmonella enterica subsp. enterica serovar Kentucky str. CVM N50429	*dnd*
Salmonella enterica subsp. enterica serovar Kentucky str. CVM N50435	*dnd*
Salmonella enterica subsp. enterica serovar Kentucky str. CVM N50437	*dnd*
Salmonella enterica subsp. enterica serovar Kentucky str. CVM N51241	*dnd*
Salmonella enterica subsp. enterica serovar Kentucky str. CVM N51249	*dnd*
Salmonella enterica subsp. enterica serovar Kentucky str. CVM N51252	*dnd*

Salmonella enterica subsp. enterica serovar Kentucky str. CVM N51256	*dnd*
Salmonella enterica subsp. enterica serovar Kentucky str. CVM N51273	*dnd*
Salmonella enterica subsp. enterica serovar Kentucky str. CVM N51277	*dnd*
Salmonella enterica subsp. enterica serovar Kentucky str. CVM N51294	*dnd*
Salmonella enterica subsp. enterica serovar Kentucky str. CVM N51313	*dnd*
Salmonella enterica subsp. enterica serovar Kentucky str. CVM N51981	*dnd*
Salmonella enterica subsp. enterica serovar Kentucky str. CVM N51982	*dnd*
Salmonella enterica subsp. enterica serovar Kentucky str. CVM29188	*dnd*
Salmonella enterica subsp. enterica serovar Kentucky str. N312	*dnd*
Salmonella enterica subsp. enterica serovar Kentucky str. SALC-205-3	*dnd*
Salmonella enterica subsp. enterica serovar Ohio CVM str. N44711	*dnd*
Salmonella enterica subsp. enterica serovar Ohio str. CFSAN001079	*dnd*
Scytonema tolypothrichoides VB-61278	*dnd*
Selenomonas sp. CM52	*dnd*
Silicibacter sp. TrichCH4B	*dnd*
Sinorhizobium meliloti 4H41	*dnd*
Streptomyces avermitilis MA-4680 = NBRC 14893	*dnd*
*Streptomyces grise*us S4-7	*dnd*
Streptomyces griseus subsp. griseus NRRL WC-3480	*dnd*
Streptomyces iakyrus NRRL ISP-5482	*dnd*
Streptomyces lividans 1326	*dnd*

Streptomyces lividans 66	*dnd*
Streptomyces lividans TK24	*dnd*
Streptomyces rimosus subsp. rimosus NRRL WC-3929	*dnd*
Streptomyces rimosus subsp. rimosus NRRL WC-3930	*dnd*
Streptomyces roseochromogenus subsp. oscitans DS 12.976	*dnd*
Streptomyces scabrisporus DSM 41855	*dnd*
Streptomyces sp. 769	*dnd*
Streptomyces sp. CNT318	*dnd*
Streptomyces sp. FxanaD5	*dnd*
Streptomyces sp. LaPpAH-202	*dnd*
Streptomyces sp. NRRL F-2305	*dnd*
Streptomyces sp. NRRL F-2580	*dnd*
Streptomyces sp. NRRL F-5650	*dnd*
Streptomyces sp. NRRL F-5702	*dnd*
Streptomyces sp. WM6391	*dnd*
Streptomyces toyocaensis NRRL 15009	*dnd*
Subdoligranulum sp. CAG:314	*dnd*
Sulfitobacter sp. 20_GPM-1509m	*dnd*
Sulfuricella denitrificans skB26	*dnd*
Sulfurospirillum barnesii SES-3	*dnd*
Synechocystis sp. PCC 7509	*dnd*
Tamlana sedimentorum JCM 19808	*dnd*
Tenacibaculum sp. 47A_GOM-205m	*dnd*
Teredinibacter turnerae 1133Y.S.0a.04	*dnd*
Teredinibacter turnerae T8513	*dnd*
Thalassospira lucentensis MCCC 1A00383 = DSM 14000	*dnd*
Thauera linaloolentis 47Lol = DSM 12138	*dnd*
Thermomonospora curvata DSM 43183	*dnd*

Thermonema rossianum DSM 10300	*dnd*
Thioalkalivibrio sp. AKL3	*dnd*
Thioalkalivibrio sp. AKL9	*dnd*
Thioflavicoccus mobilis 8321	*dnd*
Tolumonas auensis DSM 9187	*dnd*
Tolypothrix bouteillei VB521301	*dnd*
Tolypothrix campylonemoides VB511288	*dnd*
Tumebacillus flagellatus GST4	*dnd*
uncultured marine thaumarchaeote KM3_186_C08	*dnd*
Vibrio campbellii 602L	*dnd*
Vibrio metoecus OP3H	*dnd*
Acinetobacter sp. NIPH 2036	*dpt*
Bacillus cereus AH1272	*dpt*
Bacillus cereus AH1273	*dpt*
Bacillus cereus B4147	*dpt*
Bacillus cereus BAG5O-1	*dpt*
Bacillus cereus BAG5X1-1	*dpt*
Bacillus cereus BDRD-ST196	*dpt*
Bacillus cereus F837/76	*dpt*
Bacillus cereus HuA4-10	*dpt*
Bacillus cereus m1293	*dpt*
Bacillus cereus MM3	*dpt*
Bacillus cereus VD078	*dpt*
Bacillus cereus VDM022	*dpt*
Bacillus sp. B14905	*dpt*
Bacillus sp. FJAT-14578	*dpt*
Bacillus sp. UNC322MFChir4.1	*dpt*
Bacillus thuringiensis serovar huazhongensis BGSC 4BD1	*dpt*

Bacillus weihenstephanensis NBRC 101238 = DSM 11821	*dpt*
Bacillus weihenstephanensis WSBC 10204	*dpt*
Butyrivibrio sp. VCD2006	*dpt*
Carnobacterium sp. WN1359	*dpt*
Clostridium sp. CAG:221	*dpt*
Clostridium sp. CAG:265	*dpt*
Cronobacter sakazakii 680	*dpt*
Cronobacter sakazakii ATCC 29544	*dpt*
Cronobacter sakazakii NBRC 102416	*dpt*
Escherichia coli 1-176-05_S3_C1	*dpt*
Exiguobacterium sp. NG55	*dpt*
Kurthia sp. JC8E	*dpt*
Lactobacillus composti DSM 18527 = JCM 14202	*dpt*
Lactobacillus plantarum AG30	*dpt*
Paraprevotella clara YIT 11840	*dpt*
Photobacterium angustum ATCC 33977	*dpt*
Photobacterium phosphoreum ANT-2200	*dpt*
Pontibacillus litoralis JSM 072002	*dpt*
Prevotella sp. P5-125	*dpt*
Staphylococcus epidermidis NIHLM001	*dpt*
Staphylococcus epidermidis NIHLM040	*dpt*
Staphylococcus epidermidis VCU036	*dpt*
Staphylococcus simulans ACS-120-V-Sch1	*dpt*
Staphylococcus sp. HGB0015	*dpt*
Sulfurospirillum cavolei NBRC 109482	*dpt*
Vibrio parahaemolyticus CFSAN007444	*dpt*
Vibrio vulnificus VVyb1(BT3)	*dpt*
Viridibacillus arenosi FSL R5-213	*dpt*

Weissella cibaria AB3b	*dpt*
Weissella cibaria ff3PR	*dpt*
Weissella paramesenteroides ATCC 33313	*dpt*
Yersinia mollaretii 64/02	*dpt*
Yersinia pseudotuberculosis MW109-2	*dpt*

Supplementary Table 3

Strains and Plasmids used in this study

Strains and Plasmids	Description	Reference or Source
**Strains**		
Hpx^-(LC106)	As MG1655 plus Δ(*katG17*::Tn*10*)1 Δ(*katE12*::Tn*10*)1 Δ*ahpCF'* *kan*::'*ahpF*	19
MG1655	Wild type	19
Hpx^-/Dnd^+	As Hpx^- plus pJTU1238, Dnd phenotype positive, Amp^r	This study
Hpx^-/*dndC*$^-$	As Hpx^- plus pJTU3527, Dnd phenotype negative, Amp^r	This study
Hpx^-/*dndD*$^-$	As Hpx^- plus pJTU3528, Dnd phenotype negative, Amp^r	This study
Hpx^-/*dndE*$^-$	As Hpx- plus pJTU3529, Dnd phenotype negative, Amp^r	This study
E. coli WM3064	thrB1004 pro thi rpsL hsdS lacZDM15 RP4-1360 Δ(araBAD)567 ΔdapA1341::[erm pir(wt)]	47
WP3	Wild type of a deep-sea bacterium *Shewanella piezotolerans*	lab stock
WP3ΔSW1	a phage-free mutant derived from WP3，Kan^r	lab stock
WP3NR	non-restricting and phage-free mutant derived from WP3	This study
WP3NR/ Dnd^+	As WP3NR plus pSW2dnd, Dnd phenotype positive, Chl^r	This study
WP3NR/ Dnd^-	As WP3NR plus pSW2, Dnd phenotype negative, Chl^r	This study
E. coli DH10B	Non-restricting host strain for gene cloning	48
**Plasmids**		
pJTU1238	pBluescript II sk(+) derivative carrying a 6.7kb fragment of *dnd*BCDE gene cluster from S. enterica Cerro 87, with *dnd* self-promoter, Amp^r	3

pJTU3527	A derivative of pJTU1238, *dnd*C$^-$, in-frame deletion, Amp^r	[18]
pJTU3528	A derivative of pJTU1238, *dnd*D$^-$, in-frame deletion, Amp^r	[18]
pJTU3529	A derivative of pJTU1238, *dnd*E$^-$, in-frame deletion, Amp^r	[18]
pBluescript II sk(+)	E. coli cloning vector, bla, Bla, lacZ, orif1, ColE, Amp^r	Fermentas
pJTU3619	*E.coli dndA* gene cloned in pet15b	[49]
pRE112	Allelic-exchange vector for gene mutation in WP3	[50]
pRE112R1	pRE112 containing the PCR fragment for deleting a restriction endonuclease gene *swp0009*	This study
pRE112R2	pRE112 containing the PCR fragment for deleting a restriction endonuclease gene *swp840*	This study
pRE112R3	pRE112 containing the PCR fragment for deleting a restriction endonuclease gene *swp2190*	This study
pSW2	WP3 cloning vector, derivative from filamentous bacteriophage SW1, Chl^r	[51]
pSW2Dnd$^+$	pSW2 derivative carrying *dndA* from pJTU3619 and *dnd* gene cluster from pJTU 1238.	This study
